# A multi-detector chromatographic approach for characterization and quantitation of botanical constituents to enable in silico safety assessments

**DOI:** 10.1007/s00216-018-1163-y

**Published:** 2018-07-11

**Authors:** Timothy R. Baker, Brian T. Regg

**Affiliations:** The Procter & Gamble Company, 8700 Mason-Montgomery Road, Mason, OH 45040 USA

**Keywords:** *Ginkgo biloba*, High-resolution mass spectrometry, Threshold of toxicological concern, Botanical analysis, Charged aerosol detection

## Abstract

An approach has been developed to characterize the individual chemical constituents of botanicals. The challenge was to identify and quantitate the significant analytes in these complex mixtures, largely in the absence of authentic standards. The data-rich information content generated by this three-detector configuration was specifically intended to be used to conduct safety and/or quality evaluations for complex botanical mixtures, on a chemical constituent basis. The approach utilized a broad gradient UHPLC chromatographic separation. Following the chromatographic separation and UV detection, the eluent was split and sent into a charged aerosol detector (CAD), for quantitation, and a quadrupole/time-of-flight high-resolution mass spectrometer for component identification. The known bias of the otherwise universal CAD response, for organic solvent composition of the mobile phase, was compensated by the addition of an inverse gradient make-up stream. This approach and the orthogonal information content from the chromatography and three different detectors was specifically designed to enable in-silico safety assessments. These guide, minimize, or even eliminate the need for in vivo and in vitro safety assessments. The methodology was developed and demonstrated using standardized extracts of *Ginkgo biloba*. Results from the development of this novel approach and the characterization example reported here demonstrate the suitability of this instrumental configuration for enabling in-silico safety assessments and proving general quality assessments of botanicals.

**Electronic supplementary material:**

The online version of this article (10.1007/s00216-018-1163-y) contains supplementary material, which is available to authorized users.

## Introduction

Botanicals are present not only in the food chain but have also gained wide acceptance as nutritional supplements. Many botanicals are also reported to have medicinal capabilities and are part of organized practices in traditional medicine. In many cases, these botanicals have significant history of human usage and this perspective often supports safety assessments. For other botanicals, this human usage perspective is neither available nor adequate and, in those situations, additional data are required to support safety assessments.

An alternative to safety testing of the whole extract is to semi-quantitatively determine and then assess the safety of the botanical’s chemical constituents. This is one of the approaches recommended [1] for more accurate safety assessment of botanicals used in foods. The literature is replete with evidence of the improving ability of various analytical technologies to characterize complex botanical mixtures. However, quantitating the individual chemical constituents, in the absence of authentic reference standards, remains problematic.

High-performance liquid chromatography (HPLC), combined with both UV (ultraviolet) detection and high-resolution mass spectrometry (HRMS), is a well-known platform for the identification of analytes in complex mixtures. Neither detector is particularly well-suited for quantitation of various molecules across or often even within compound classes, in the absence of authentic reference standards. The UV detector is dependent on the presence of a chromophore in the molecules of interest and their ability to absorb UV (and visible) light. This can obviously vary from structure-to-structure and in particular across compound classes. The ionization/volatilization interface of an HPLC/MS system is even more variable and is sensitive to even small changes in molecular structure. Therefore, while useful for identification of unknowns, neither of these detectors is suitable for providing quantitation data for a complex mixture containing constituents for which reference standards are not available. This requires a general detector where signal is directly proportional to analyte mass, in much the same way that flame ionization detection (FID) is utilized when coupled with gas chromatography [2]. Quantitation of natural product constituents has been demonstrated with quantitative nuclear magnetic resonance [3]; however, the approach is not suitable for comprehensive quantitation of complex mixtures containing many analytes with similar structural features.

A charged aerosol detector (CAD) is suitable for detection of semi-volatile and non-volatile analytes (boiling point > 350 °C) separated by HPLC. This detector provides signal that corresponds to the mass of each analyte, irrespective of chemical structure [4].

We have assembled an instrumental configuration to separate complex botanical mixtures using ultra-high-performance liquid chromatography (UHPLC) followed by UV, CAD, and HRMS detection. This allows direct correlation between identification and relative quantitation of chemical constituents in complex mixtures. The experiment has been optimized specifically to produce data that is then utilized in threshold of toxicological concern [1] and other in-silico safety assessments, as well as quality evaluations. A fairly well-characterized botanical, *Ginkgo biloba*, was chosen to develop and illustrate this approach [5–10].

## Materials and methods

### Sample preparation

The *Ginkgo biloba* leaf extract used was Spectrum Ginkgo Standardized Extract (Lot # YT0930, Spectrum Chemical, 769 Jersey Ave., New Brunswick, NJ 08901-3605). The extract, a dry powder, was stored at room temperature, the recommended use condition, for the duration of the study (several months). The definition of “standardized extract” is flavonoid glycosides > 24% and triterpene lactones > 6%. The methods the vendor used to support these claims is not known to us; however, these numbers correlate with our results. The extract powder was dissolved in 70/30 ethanol/water to 20 mg/mL, vortexed for 30 min, and then filtered through a 0.2 μm PTFE membrane.

### Reference standards

The standards were reference materials (not certified reference materials) with purities greater than or equal to 95%. Standards were prepared at 25 ng/μL in 70% ethanol/30% water. In all cases, the accurate MS and MS/MS data supported that the standards were as labeled. The standards were obtained from several sources. Chromadex (Irvine, CA): Xanthurenic acid, Isorhamnetin, Isoquercetin, Kaempferol rutinoside, Isorhamnetin glucoside, Genistein, Kaempferol. Sigma-Aldrich Corp. (St. Louis, MO): l-Proline, l-Valine, Choline, Mannitol, l-Tryptophan, Caffeine, Catechin, Gallocatechin, Vanillic Acid, Esculetin, Ginkgolide A, Ginkgolide B, Ginkgolide C, Ginkgolide J, Rutin, Quercetin, Bilobalide, Quercitrin, Isorhamnetin rutinoside, Soyasaponin I, Ginkgolic Acid (C13:0), Ginkgolic Acid (C15:1); MP Biomedicals (Solon, OH): Protocatechuic acid, Myricetin; Alfa Aesar Tewksbury, MA)—Salicylic acid, Quinic acid; TCI (Portland OR)—Trigonelline.

### Instrumental conditions

**Fig. 1:**
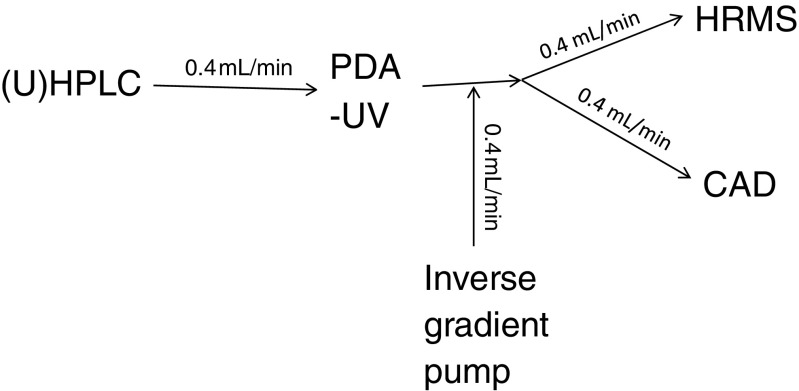
Schematic of the instrumental configuration for UHPLC/UV/CAD/Q-TOF MS experiments

The instrumental configuration for this method is shown in Fig. 1. Essential experimental details are shown below, with additional details provided in the Electronic Supplementary Material (ESM) Table S2.

#### Chromatography

An Agilent Technologies (5301 Stevens Creek Blvd Santa Clara, CA 95051 USA) 1290 Infinity chromatograph (UV photodiode array detector (PDA)) was used for the UHPLC separations, with a Waters (34 Maple Street, Milford, MA 01757 USA) Acquity BEH C_18_ (2.1 × 150 mm, 1.7 μm particles) column. Injections used 3 μL of sample extracts. The separation used mobile phase A (water with 0.1% formic acid (FA)) and B (acetonitrile with 0.085% FA) with a flow rate of 0.4 mL/min. The gradient consisted of a 5-min hold at 2% A, followed by a linear ramp from 2% A to 100% A, at 1%/min, for a total acquisition time of 125 min.

#### Mass spectrometry

The high-resolution mass spectrometry detection was performed using an Agilent 6540 UHD Accurate Mass quadrupole-time-of-flight (Q-TOF) system, with MassHunter software (version B.04.00). Experiments were conducted in positive and negative ion modes, using the Agilent Jetstream**®** electrospray ionization (ESI) and atmospheric pressure chemical ionization (APCI) sources. Mass spectrometry data was collected from *m*/*z* 50–3200 at 5 Hz. Tandem mass spectrometry (MS/MS) experiments, specifically product ion scans, were performed on [M + H]^+^, [M − H]^−^ or other precursor ions.

#### Quantitation detector

A charged aerosol detector from ESA (now part of Thermo Scientific, 81 Wyman Street, Waltham, MA 02451 USA) was used to quantitate the analytes. This detector signal output was connected to the mass spectrometer data system through an Agilent 35900E interface (analog-to-digital converter). Additionally, the gas used was N_2_ at 35 PSI, with a detector range of 100 pA and a data collection rate of 10 Hz.

#### Inverse gradient

A separate HPLC pump was utilized to deliver the inverse gradient to the flowing stream, post-column (see Fig. 1). A Shimadzu (1, Nishinokyo-Kuwabaracho, Nakagyo-ku, Kyoto 604-8511, Japan) controller (SCL-10A) and pumps (LC-10AD), with a Gilson (3000 Parmenter Street, Middleton, WI 53562 USA) 811C Dynamic mixer (65 μL), was used for this purpose, triggered by a contact closure connection to the mass spectrometer data system, via the Agilent UV detector.

## Results and discussion

The instrumental configuration shown in Fig. 1 was intended to optimize the separation of complex mixtures and gather information from three separate detectors in order to most easily correlate identifications made by the mass spectrometer with quantitation by the CAD. Separate experiments using the mass spectrometer and CAD are problematic due to the need to very carefully correlate the presence of multiple analytes, observed by MS, within CAD peaks and more generally by the complex nature of these mixtures. In practice, the analysis of a sample was performed multiple times to gather different mass spectrometry data, positive and negative ion data, compare ESI and APCI volatilization/ionization modes, and then to perform MS/MS experiments to aid identifications on analytes of interest, but in each case by carefully correlating UV, MS, and CAD data. This approach allows the collection of orthogonal data sets on the sample. These are chromatographic retention, UV spectra, quantitative CAD signals, and a variety of MS and MS/MS spectra. There were a number of experimental considerations that facilitated data generation intended to support the in-silico safety assessments.

### Chromatography

The primary considerations in chromatographic method development are to separate the individual constituents to the extent possible and elute all the analytes present in the sample. Furthermore, sample numbers in these experiments are low and data acquisition consumes less time than data mining and interpretation. Therefore, longer empirically developed chromatographic separations, such as the 125 min one shown here, that pull similar analytes apart and ideally present them to the detectors individually are the objective. The separation here started with very low organic content (2%) to 100% organic, with a slow gradient, in order to elute a broad range of analytes (highly polar to non-polar). In practice, it has, in our experience, never been possible to fully separate the many analytes in a botanical extract; however, optimizing the separation of the overall mixture does facilitate data interpretation and quantitation. UHPLC is preferred whenever possible, over HPLC, due to its superior resolving capability, but there are cases when the preferred stationary phase may be available only in an HPLC format (e.g., for botanicals with significant percentages of relevant highly polar, early eluting analytes). The highly polar analytes that are poorly separated in the first 5 min of the example analysis here are of less interest or concern with respect to the safety assessment; therefore, chromatographic conditions more suitable to medium polar analytes were used.

### Inverse gradient

**Fig. 2:**
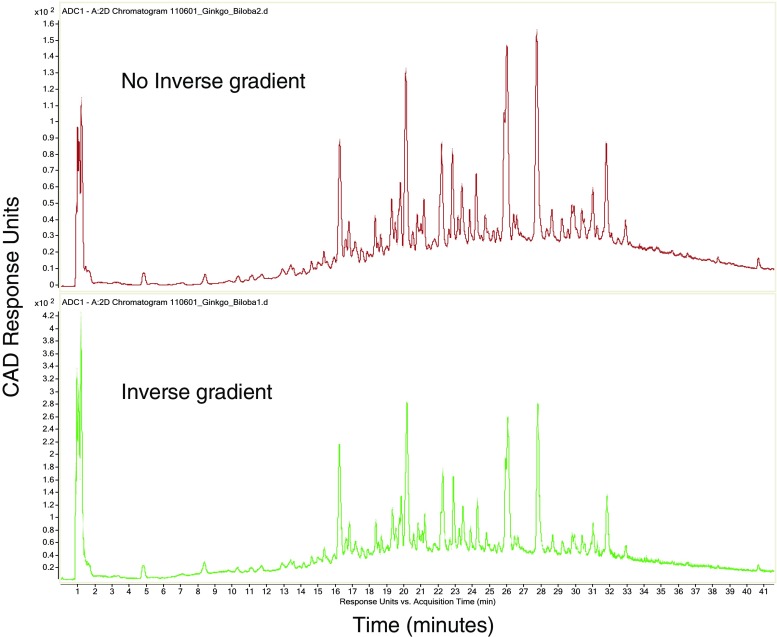
Post-column addition of gradient running inverse to the eluent consistently presented a constant ~50:50 acetonitrile:water mixture to the charged aerosol detector, reducing the known bias of CAD response relative to organic composition. Partial chromatograms shown, illustrating improvements in the early (high aqueous) portion of the analysis (note peak height v. signal intensity on y-axis). Total run time was 125 min

**Fig. 3:**
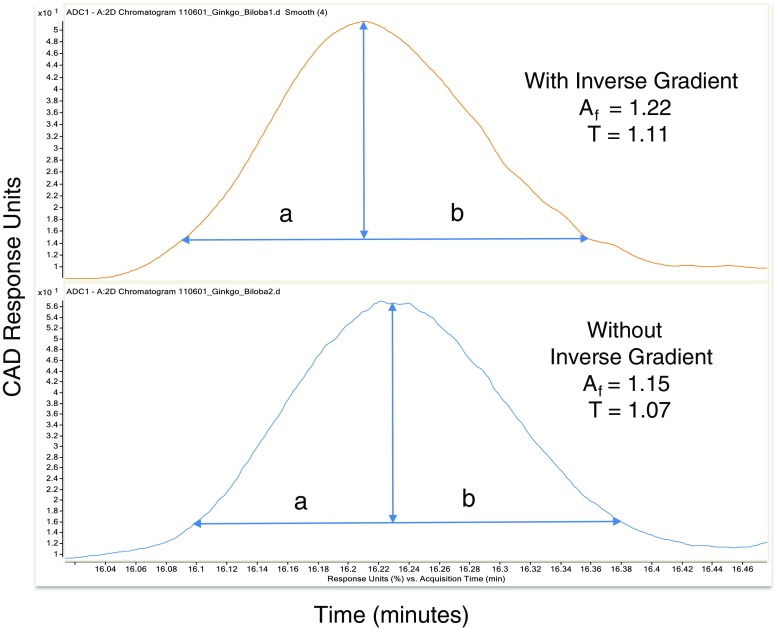
Illustration that the addition of the inverse gradient did not appreciably broaden the chromatographic peaks, shown here for Bilobilide. The USP system suitability factors were used: Peak asymmetry factor *A*_f_ = b_10% h_ /a_10% h,_ Tailing factor *T* = (a_5%h_ + b_5%h_)/2a_5%h_

This inverse gradient addition mirrored the UHPLC gradient such that the CAD (and mass spectrometer) were presented with a consistent ~50/50 organic/water mixture. This served to eliminate the known response bias of the CAD (less responsive to high aqueous content) and maintained a consistent response across the entire analysis [11, 12]. In our hands, the intensity difference between the same analyte detected via flow injection/CAD analysis in 100% aqueous and then in 100% organic varied by as much as sixfold (data not shown). Furthermore, the addition of organic content by this inverse gradient arrangement served to increase both the CAD and MS response for the majority of polar analytes that eluted in the relatively early, high aqueous portion of the separation. The inverse gradient increased the CAD signal by a factor of two or three in the majority of the chromatogram (Fig. 2). More importantly, from a chromatographic perspective, the inverse gradient did not have an adverse effect on peak shape, as noted by comparing peak asymmetry and tailing factors (Fig. 3).

### Charged aerosol detection

With the ultimate objective, in this work, of a safety assessment of the constituents at or above the threshold of toxicological concern, attempts to identify analytes, with the other detectors, were keyed off CAD peaks observed (quantitative data). To maximize the dynamic range of the experiment and quantitate as many analytes as possible, the amount of sample injected on-column can be adjusted such that the most abundant CAD peak is just short of saturation. Then, CAD peaks as low as 0.05% of the total CAD signal can be observed, with a signal-to-noise ratio of at least 3:1. Analytes not observed by CAD but identified by MS can then be indicated as < 0.05% of the mass of the sample, in this case. Analytes were quantitated based upon percentage of the overall CAD signal. While this is not the most accurate means of quantitation and could be called semi- or relative-quantitation, it is possible in the absence of standards of each analyte and is adequate for the purposes of the inherently conservative threshold of toxicological concern assessment. Preliminary comparison of the CAD quantitation to quantitation with standards of compounds used for positive identifications suggests CAD accuracy is no worse than a factor of 2, again, adequate for these purposes. That said, an approach using response factors with CAD signal promises significantly better accuracy. Investigations in progress will define accuracy and precision more completely, in a future report. Alternately, evaporative light scattering detection (ELSD) could be used in a similar manner; however, in our hands and in literature reports [13, 14], this technology has less sensitivity than CAD.

### High-resolution mass spectrometry

HRMS is vital to the confident identification of botanical constituents, particularly in the absence of standards. HRMS can be utilized to quickly assign molecular formula. Searching a formula (e.g., with SciFinder) in the context of a particular botanical often provides feasible results [15]. MS/MS experimentation with accurate mass results provides structural clues than can support these search results, be compared against fragmentation of other molecules within the same class, or even provide identification when compared to databases such as mzCloud [16].

### Characterization approach

The identifications reported here were made with the following important considerations.

- No attempt to fully characterize or exhaustively identify all the components of the extract was made. Rather, identification of the compounds that gave a detectable CAD response in these particular analyses was attempted. The amount of material analyzed was adjusted to provide CAD signals down to the threshold of toxicological concern [17]. Although the mass spectrometer could often detect analytes below this level, these constituents were not considered because the in silico safety assessment was the goal of this work.
- It is possible that organic components of the samples did not elute from the column or respond to either UV or CAD detection, electrospray, or atmospheric chemical ionization and were therefore not observed. Volatile analytes in particular would not be expected to be detected using this approach; however, in this case, the sample was dried powder and where therefore unlikely to contain a significant amount of volatile material.
- The isotope fitting “score” (shown in ESM Table S1) is a software calculation (called MFG Score in the software), from 0 to 100, that compares the theoretical data for the given empirical formula to the experimental data. The score calculation factors in comparison of mass accuracy, abundance fit of naturally occurring isotope ratio (the monoisotopic peak A, compared to A + 1 and A + 2), and exact mass spacing of *m*/*z* peaks in the isotopic envelope. A score above 90 is considered confident and in most cases the assigned molecular formula had the highest score of the possible formulae. In some instances, lower scores or mass accuracies do not indicate an incorrect assignment, but were caused by interferences from chemical background, co-eluting analytes, low level signal, or experimental variance. A sense of how well the mass accuracy and scores work can be obtained from the values determined for those analytes that were confirmed by authentic standard comparison (i.e., known to be correct).
- In many cases, authentic standards of the compounds were also analyzed, when available, and this allowed definitive identification of these compounds. In all other cases, the proposed identifications were made with variable confidence (dimensioned in the table), based upon the data, expectations of what should be present from the literature and comparison to mass spectral data of authentic standards of similar compounds.

### Test case results

**Fig. 4:**
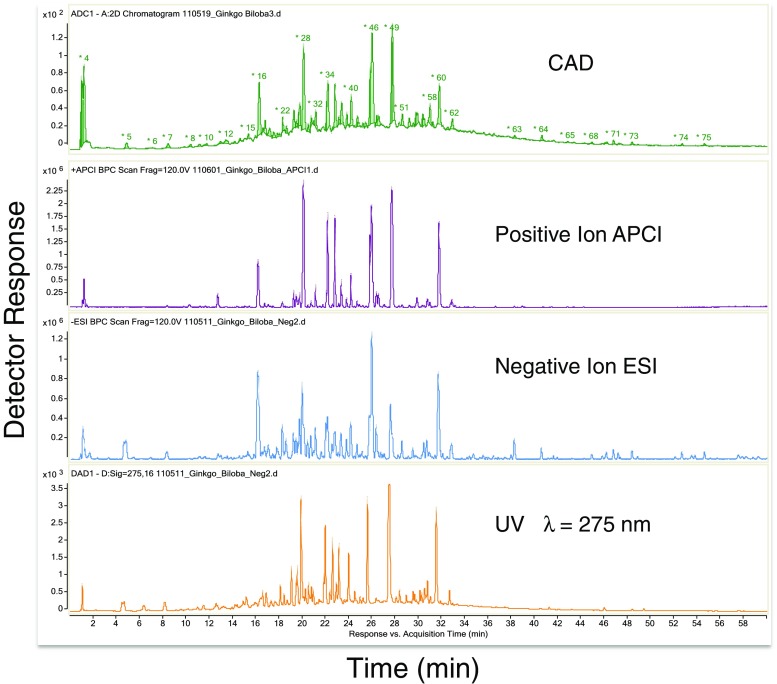
Partial chromatograms from the UHPLC/UV/CAD/Q-TOF-MS analysis of the Ginkgo extract showing the majority of analytes from the 125-min analysis. Note peak numbers on the CAD chromatogram. Results in Table 1 are organized by peak number

The chromatographic results from the analysis of the standardized *Ginkgo biloba* extract are shown in Fig. 4. Partial chromatograms are shown to best illustrate the majority of analytes observed by the three detectors (UV, CAD, HRMS), and two MS ionization/volatilization modes (electrospray (ESI) and atmospheric chemical ionization (APCI)). CAD, UV, and MS results from only one ionization/volatilization mode could be obtained in a single experiment. Separate analyses were required to obtain data in additional MS modes or to obtain MS/MS information (data-dependent analysis not used here).

Relative amounts of 83 peaks, down to 0.05% of the total CAD signal, were obtained by CAD, for this botanical extract. UHPLC resolved isomers of known analytes and presented fewer coeluting peaks to the detectors than was possible via HPLC. The mass spectrometer was found to be more sensitive than the CAD, as expected. Indications of a least 50 lower level analytes not seen by CAD (< 0.05% of total signal each) or coeluting in CAD peaks as minor signals observed by mass spectrometery were noted but not characterized or interpreted. Quantitation and identification of these lower level analytes were not pursued as they fell below the level of interest (i.e., threshold of toxicological concern). Data were collected in the APCI mode in case some analytes did not respond via ESI; however, no unique data were obtained by APCI, even for late-eluting, presumably non-polar analytes (peaks 79, 82, and 83). For the purposes of this preliminary investigation, the broad hump in the chromatogram that the majority of the peaks are sitting on, which was not observed in the blank, has not been factored into consideration. This is believed to have been caused by chromatographically unresolved tannins [18]. The presumed tannin hump is not observed in the mass spectrometry chromatograms shown in Fig. 4 as these are from lower amount injections required to keep the other analytes on scale.

The results of the characterizations of the 83 CAD peaks are shown in Table 1. The identifications are presented in elution order and peak numbers correspond to those shown in the CAD chromatogram in Fig. 4. In cases where an analyte has been identified with high confidence (i.e., matched to an authentic standard), the name and chemical abstracts service (CAS) number are provided (from the material containers). CAS numbers for other known compounds were obtained from SciFinder, recognizing that these numbers can vary based upon salt form, synthetic source, and that some analytes have multiple CAS numbers. Additionally, comments are made for each peak’s identification to indicate the level of confidence in the proposed structure and other information that may be of relevance to a safety assessment (e.g., the presence of specific functional groups) [17]. For the purposes of a safety assessment using threshold of toxicological concern, which focuses on comparing functional groups and substructure of molecules, absolute identification of a molecule and its specific connectivity are not essential. An identification of a molecule that includes its functionalities, but not all the connectivity, is still useful. Even a partial identification, such as the presence of glycosylation, has value when the identified substructure is considered detoxifying.

**Table 1:** Proposed identifications of the 83 CAD peaks observed during the analysis of the Spectrum Ginkgo extract

CAD Peak number	Proposed ID (CAS #)	Molecular Formula	Comments
1	Magnesium	Mg^2+^	Magnesium clusters with acetonitrile and formic acid in positive ion mode and Magnesium clusters with formic acid in negative ion mode. Example here is [MgFm+2ACN]^+^. MgFm2 repeat has a mass difference of 113.98043. In negative ion, example is [Mg+3Fm]^-^. Isotope pattern consistent with Mg.
2	Choline (67-48-1)	C_5_H_14_N_1_O_1_	RT, UV and MS/MS spectra consistent with authentic standard.
3	Trigonelline (6138-41-6)	C_7_H_7_N_1_O_2_	RT, UV and MS/MS spectra consistent with authentic standard.
	L-Proline (147-85-3)	C_5_H_9_N_1_O_2_	RT, UV and MS/MS spectra consistent with authentic standard.
	L-Valine (72-18-4)	C_5_H_11_N_1_O_2_	RT, UV and MS/MS spectra consistent with authentic standard.
	Mannitol (69-65-8)	C_6_H_14_O_6_	RT, UV and MS/MS spectra consistent with authentic standard.
	Quinic Acid (77-95-2)	C_7_H_12_O_6_	RT, UV and MS/MS spectra consistent with authentic standard.
4	Ginkgotoxin-5-O-glucoside (323579-25-5)	C_15_H_23_N_1_O_8_	MS/MS indicates hexose and is consistent with structure [20].
	Unknown	C_7_H_12_O_5_	Unknown, but probably similar to Quinic acid (less oxygen).
5	Protocatechuic Acid (99-50-3)	C_7_H_6_O_4_	RT, UV and MS/MS spectra consistent with authentic standard.
6	Xanthurenic Acid (59-00-7)	C_10_H_7_N_1_O_4_	RT, UV and MS/MS spectra consistent with authentic standard.
7	Unknown	C_16_H_24_O_9_	Observed as [M+NH_4_]^+^ in positive ion mode and [M+ Formate]^-^ in negative ion mode.
	L-Tryptophan (73-22-3)	C_11_H_12_N_2_O_2_	RT, UV and MS/MS spectra consistent with authentic standard.
8	Caffeine (58-08-2)	C_8_H_10_N_4_O_2_	RT, UV and MS/MS spectra consistent with authentic standard.
9	Gallocatechin (3371-27-5)	C_15_H_14_O_7_	RT, UV and MS/MS spectra consistent with authentic standard.
	Hydroxy-benzaldehyde	C_7_H_6_O_2_	MS/MS spectrum suggests Hydroxybenzaldehyde
10	Vanillic Acid (121-34-6)	C_8_H_8_O_4_	RT, UV and MS/MS spectra consistent with authentic standard.
	Esculetin (305-01-1)	C_9_H_6_O_4_	RT, UV and MS/MS spectra consistent with authentic standard.
	Catechin (7295-85-4)	C_15_H_14_O_6_	RT, UV and MS/MS spectra consistent with authentic standard.
	Salicylic Acid (69-72-7)	C_7_H_6_O_3_	RT, UV and MS/MS spectra consistent with authentic standard.
	Quercetin with 2 glucose and 1 rhamnose	C_39_H_50_O_25_	MS/MS shows losses of glucose and rhamnose.
11	Unknown	C_19_H_28_O_11_	Observed as [M+NH_4_]^+^ in positive ion mode and [M+ Formate]^-^ in negative ion mode. MS/MS indicates hexose present.
12	Unknown	C_32_H_44_O_17_	Observed as [M+NH_4_]^+^ in positive ion mode and [M+ Formate]^-^ in negative ion mode. MS/MS indicates hexose present.
13	Kaempferol tetraglycoside (2 glucose and 2 rhamnose)	C_39_H_50_O_24_	Proposed compound from MS/MS data. Connectivity unknown.
14	Glucopyranosyl rutin	C_33_H_40_O_21_	Proposed compound based on MS/MS data.
	Astilbin (29838-67-3)	C_21_H_22_O_11_	Proposed based on MS/MS data.
15	Kaempferol rhamnosyl glucoside	C_27_H_30_O_15_	Proposed compound based on MS/MS data and literature [21]. Observed as [M+ Formate]^-^ in negative ion mode.
16	Bilobalide (33570-04-6)	C_15_H_18_O_8_	RT, UV and MS/MS spectra consistent with authentic standard.
17	Quercetin rhamnosyl rutinoside isomer	C_33_H_40_O_20_	Proposed compound based on MS/MS and literature [21].
18	Kaempferol di-rhamnosyl- glucoside	C_33_H_40_O_19_	Proposed structure based on MS/MS and literature [21].
	Isorhamnetin di-glucosyl- rhamnoside	C_34_H_42_O_21_	Proposed structure based on MS/MS.
19	Unknown	C_15_H_20_O_9_	This unknown one of several analytes within this peak. *
20	Quercetin rhamnosyl rutinoside isomer	C_33_H_40_O_20_	Proposed compound based on MS/MS.
	Unknown	C_27_H_30_O_15_	MS/MS shows some indication of a possible kaempferol rutinoside.
	Kaempferol glucosyl-coumaryl-glucosyl rhamnoside	C_48_H_56_O_27_	MS/MS fragments indicate loss of coumaryl, rhamnose and hexose.
21	Myricetin rutinoside	C_27_H_30_O_17_	Proposed structure based on MS/MS and literature [22].
	Bilobalide isomer	C_15_H_18_O_8_	Probable isomer of Peak 16.
22	Quercetin rhamnosyl rutinoside isomer	C_33_H_40_O_20_	Proposed compound based on MS/MS and literature [21].
23	Quercetin rhamnosyl rutinoside isomer	C_33_H_40_O_20_	Proposed compound based on MS/MS and literature [21].
24	Rutin isomer	C_27_H_30_O_16_	Proposed compound based on MS/MS and literature [22].
25	Ginkgolide J (107438-79-9)	C_20_H_24_O_10_	RT, UV and MS/MS spectra consistent with authentic standard. Observed as [M+ Formate]^-^ in negative ion mode.
26	Quercetin glucosyl-coumarylglucosyl rhamnoside	C_42_H_46_O_23_	Proposed structure based on MS/MS and literature [22].
27	Kaempferol di-rhamnosyl- glucoside	C_33_H_40_O_19_	Proposed structure based on MS/MS. Kaempferol fragment ion observed in MS/MS [21].
28	Rutin (153-18-4)	C_27_H_30_O_16_	RT, UV and MS/MS spectra consistent with authentic standard. Possible second isomer nearly coeluting with Rutin.
	Ginkgolide C (15291-76-6)	C_20_H_24_O_11_	RT, UV and MS/MS spectra consistent with authentic standard. Relative amounts of these two coeluting compounds probably not different by more than a factor of two.
29	Isoquercetin (482-35-9)	C_21_H_20_O_12_	RT, UV and MS/MS spectra consistent with authentic standard.
	Laricitrin rutinoside Isomer	C_28_H_32_O_17_	Proposed structure based on MS/MS and literature [22]. Isorhamnetin fragment observed in MS/MS spectrum.
30	Laricitrin rutinoside Isomer	C_28_H_32_O_17_	Proposed structure based on MS source fragments, literature [22].
31	Kaempferol glucosylcoumarylglucosyl rhamnoside	C_42_H_46_O_22_	Proposed structure based on MS/MS and literature [22].
32	Quercetin glucosylcoumarylglucosyl rhamnoside	C_42_H_46_O_23_	Proposed structure based on MS/MS and literature [22].
	Unknown	C_26_H_34_O_11_	Observed as [M+NH_4_]^+^ in positive ion mode and [M+ Formate]^-^ in negative ion mode. MS/MS indicates hexose present.
33	Isomer of Rutin	C_27_H_30_O_16_	Structure similar to Rutin based on similar MS/MS fragment ions observed from Rutin. MS/MS spectrum shows aglycone quercetin fragment.
34	Kaempferol rutinoside (17650-84-9)	C_27_H_30_O_15_	RT, UV and MS/MS spectra consistent with authentic standard.
35	Quercitrin (522-12-3)	C_21_H_20_O_11_	RT, UV and MS/MS spectra consistent with authentic standard.
36	Isorhamnetin rutinoside (604-80-8)	C_28_H_32_O_16_	RT, UV and MS/MS spectra consistent with authentic standard.
37	Limocitrin rutinoside (489-33-8)	C_29_H_34_O_17_	Possibly Limocitrin (489-33-8) rutinoside. MS/MS indicates rutinoside and limocitrin aglycone.
	Kaempferol glucoside isomer	C_21_H_20_O_11_	MS/MS indicates presence of hexose.
	Myricetin (529-44-2)	C_15_H_10_O_8_	RT, UV and MS/MS spectra consistent with authentic standard.
	Unknown	C_26_H_46_O_14_	MS/MS indicates hexose present.
38	Genistein rutinoside	C_27_H_30_O_14_	MS/MS suggests genistein aglycone, hexose and rhamnose (cannot rule out apigenin as the aglycone). This appears to be the more abundant of the two similar coeluting compounds.
	Isorhamnetin glucoside (5041-82-7)	C_22_H_22_O_12_	RT, UV and MS/MS spectra consistent with authentic standard.
39	Unknown	C_28_H_36_O_13_	Observed as [M+NH_4_]^+^ in positive ion mode. MS/MS indicates presence of hexose.
40	Isomer of Kaempferol rutinoside (17650-84-9)	C_27_H_30_O_15_	MS/MS suggests hexose and kaempferol aglycone.
	Kaempferol rutinoside substructure	C_43_H_46_O_22_	MS/MS data confirms substructure of kaempferol rutinoside. Remainder of formula corresponds to epigallocatechin methyl ether.
41	Isomer of Quercitrin (522-12-3)	C_21_H_20_O_11_	MS/MS confirms quercetin aglycone and rhamnose.
	Unknown	C_26_H_34_O_10_	Observed as [M+NH_4_]^+^ in positive ion mode and [M+ Formate]^-^ in negative ion mode.
42	Unknown	C_32_H_42_O_14_	This empirical formula is confident, but MS/MS does not clarify structure.
43	Kaempferol rhamnoside	C_21_H_20_O_10_	MS/MS confirms kaempferol aglycone and rhamnose.
	Unknown	C_20_H_36_O_11_	Observed as [M+NH_4_]^+^ in positive ion mode and [M+ Formate]^-^ in negative ion mode. MS/MS confirms hexose.
44	Ginkgolide Isomer	C_20_H_24_O_10_	Possibly Ginkgolide M. Also observed as [M+NH_4_]^+^ in positive ion mode and [M+ Formate]^-^ in negative ion mode. MS/MS spectrum similar to that of isobaric Ginkgolide B (Peak 46)
45	Quercetin coumarylglucosyl rhamnoside isomer	C_36_H_36_O_18_	Assignment based on literature [22] and MS/MS in negative ion mode. MS/MS suggests coumaryl group, rutinoside and quercetin.
46	Ginkgolide A (15291-75-5)	C_20_H_24_O_9_	RT, UV and MS/MS spectra consistent with authentic standard. Also observed as [M+NH_4_]^+^ in positive ion mode and [M+ Formate]^-^ in negative ion mode.
	Ginkgolide B (15291-77-7)	C_20_H_24_O_10_	RT, UV and MS/MS spectra consistent with authentic standard. Also observed as [M+NH_4_]^+^ in positive ion mode and [M+ Formate]^-^ in negative ion mode.
47	Unknown	C_21_H_30_O_9_	Observed as [M+NH_4_]^+^ in positive ion mode and [M+ Formate]^-^ in negative ion mode. MS/MS indicates hexose.
48	Unknown	C_19_H_22_O_5_	MS/MS shows facile loss of formic acid.
49	Quercetin (117-39-5)	C_15_H_10_O_7_	RT, UV and MS/MS spectra consistent with authentic standard.
50	Kaempferol coumarylglucosyl rhamnoside isomer	C_36_H_36_O_17_	MS/MS confirms coumaryl, rhamnosyl hexosyl and kaempferol aglycone. Assignment also based on literature [22].
51	Quercetin coumarylglucosyl rhamnoside isomer	C_36_H_36_O_18_	MS/MS confirms coumaryl, rhamnosyl hexosyl and quercetin aglycone. Assignment also based on literature [22].
52	Kaempferol-quercetin coumarylglucosyl rhamnoside isomer	C_72_H_72_O_35_	Appears to be chromatographically distinct dimer of MW = 740 and MW = 756 compound (Peaks 50 and 51). Observe doubly charged ion in both negative and positive ion modes. MS/MS confirms kaempferol and quercetin aglycones, rhamnose, glucose
53	Unknown	C_21_H_34_O_9_	Proposed elemental formula based on negative ion data. MS/MS indicates hexose.
54	Kaempferol-quercetin coumarylglucosyl rhamnoside isomer	C_72_H_72_O_35_	Appears to be chromatographically distinct dimer of MW = 740 and MW = 756 compound (Peaks 50 and 51). Observe doubly charged ion in both negative and positive ion modes. MS/MS confirms kaempferol and quercetin aglycones, rhamnose, glucose
55	Kaempferol-quercetin coumarylglucosyl rhamnoside isomer	C_72_H_72_O_35_	Appears to be chromatographically distinct dimer of MW = 740 and MW = 756 compound (Peaks 50 and 51). Observe doubly charged ion in both negative and positive ion modes. MS/MS confirms kaempferol and quercetin aglycones, rhamnose, glucose
56	Kaempferol coumarylglucosyl rhamnoside isomer dimer	C_72_H_72_O_34_	Appears to be chromatographically distinct dimer of MW = 740 (Peak 50). Observe doubly charged ion in both negative and positive ion modes. MS/MS confirms kaempferol aglycone, rhamnose, hexose.
57	Kaempferol coumarylglucosyl rhamnoside isomer	C_36_H_36_O_17_	MS/MS confirms coumaryl, rhamnosyl hexosyl and kaempferol aglycone. Assignment also based on literature [22].
58	Genistein (446-72-0)	C_15_H_10_O_5_	RT, UV and MS/MS spectra consistent with authentic standard.
59	Kaempferol coumarylglucosyl rhamnoside isomer dimer	C_72_H_72_O_34_	Appears to be chromatographically distinct dimer of MW = 740 (Peak 50). Observe doubly charged ion in both negative and positive ion modes. MS/MS confirms kaempferol aglycone, rhamnose, hexose.
60	Kaempferol (520-18-3)	C_15_H_10_O_6_	RT, UV and MS/MS spectra consistent with authentic standard.
61	Kaempferol coumarylglucosyl rhamnoside isomer dimer	C_72_H_72_O_34_	Chromatographically distinct dimer of MW = 740 (Peak 50). Observe doubly charged ion in both negative and positive ion modes. MS/MS confirms kaempferol aglycone, rhamnose, hexose.
62	Isorhamnetin (480-19-3)	C_16_H_12_O_7_	RT, UV and MS/MS spectra consistent with authentic standard.
	Kaempferol coumarylglucosyl rhamnoside isomer dimer	C_72_H_72_O_34_	Appears to be chromatographically distinct dimer of MW = 740 (Peak 50). Observe doubly charged ion in both negative and positive ion modes. MS/MS confirms kaempferol aglycone, rhamnose, hexose.
	Unknown	C_21_H_32_O_8_	Observed as [M+NH_4_]^+^ in positive ion mode and [M+ Formate]^-^ in negative ion mode.
63	Unknown	C_16_H_32_O_4_	Water loss fragment in positive ion mode suggest hydroxylated C16 chain acid.
64	Unknown	C_48_H_76_O_18_	Observe [M+NH4]^+^ in positive ion mode. DBE = 11. Appears to be triterpenoid saponin related structure. Possibly Dehydrosoyasaponin I.
65	Unknown	C_15_H_22_O_3_	MS/MS response too weak. No MS/MS fragments.
66	(Z)-Alpha-Atlantone (56192-70-2)	C_15_H_22_O_1_	Proposed compound based on literature references and consistent with MS/MS spectrum [22].
	Unknown	C_47_H_74_O_17_	Observed as [M+NH_4_]^+^ in positive ion mode. Appears to be triterpenoid saponin related structure.
67	Unknown	C_21_H_40_O_7_	Also observed [M+Na]^+^ and [M+NH_4_]^+^ in positive ion mode and observed [M+ Formate]^-^ in negative ion mode.
68	Soyasaponin I (51330-27-9)	C_48_H_78_O_18_	RT, UV and MS/MS spectra consistent with authentic standard.
69	Unknown	C_42_H_68_O_14_	Similar structure to Peak 68 based on MS/MS. Appears to be triterpenoid saponin related structure.
70	Unknown	C_42_H_68_O_14_	Similar structure to Peak 69 based on MS/MS. Appears to be triterpenoid saponin related structure.
71	Unknown	C_48_H_78_O_17_	Observe [M+NH4]^+^ in positive ion mode. DBE = 10. Appears to be triterpenoid saponin related structure.
72	Trihydroxy-octadecenoic acid	C_18_H_34_O_5_	MS/MS clearly suggests presence of acetate and three aliphatic hydroxyl groups. Position of hydroxyl groups and double bond unknown.
73	Unknown	C_42_H_68_O_13_	Observe [M+NH4]^+^ in positive ion mode. Appears to be triterpenoid saponin related structure.
74	Unknown	C_25_H_40_O_5_	MS/MS suggests coumaryl group and dihydroxy aliphatic C16 acid.
75	Unknown	C_33_H_56_O_14_	Observe [M+NH4]^+^ in positive ion mode and [M+ Formate]^-^ in negative ion mode.
76	Unknown	C_18_H_30_O_2_	MS/MS suggests dihydroxy- aliphatic C18 chain.
77	Unknown	C_30_H_48_O_1_	Possible triterpene compound with single hydroxyl group. Very weak. MS/MS signal.
78	Unknown	C_18_H_32_O_2_	Low Score caused by mass interferences and low signal. Signal too weak for MS/MS. Possibly Linoleic acid.
79	Unknown	Unknown	No discernable MS signal obtained for CAD peak.
80	Ginkgolic Acid (C13:0) (20261-38-5)	C_20_H_32_O_3_	RT, UV and MS/MS spectra consistent with authentic standard.
81	Ginkgolic Acid (C15:1) (22190-60-7)	C_22_H_34_O_3_	RT, UV and MS/MS spectra consistent with authentic standard.
82	Unknown	Unknown	No discernable MS signal obtained for CAD peak.
83	Unknown	Unknown	No discernable MS signal obtained for CAD peak.

*Mass Spectra from these CAD peaks also contained other lower level signals that indicate the presence of other analytes not reported

An expanded version of Table 1 is shown in ESM Table S1. This also contains the MS ionization mode data for each analyte. Some analytes were observed by both negative and positive ion MS. The mass accuracy in parts-per-million (ppm) is listed along with the score (comparison of experimental to theoretical mass accuracy and isotope fitting). In each case, the percentage of a peak’s signal relative to the total CAD signal obtained from the analysis is listed. When known, the structures of the analytes are also shown in ESM Table S1. In some cases, connectivity of groups could not be determined and Markush or parenthetical structures are shown, indicating known functional groups with unknown connectivity. Isomeric functional groups such as glucosides and galactosides could not be differentiated by this approach.

**Fig. 5:**
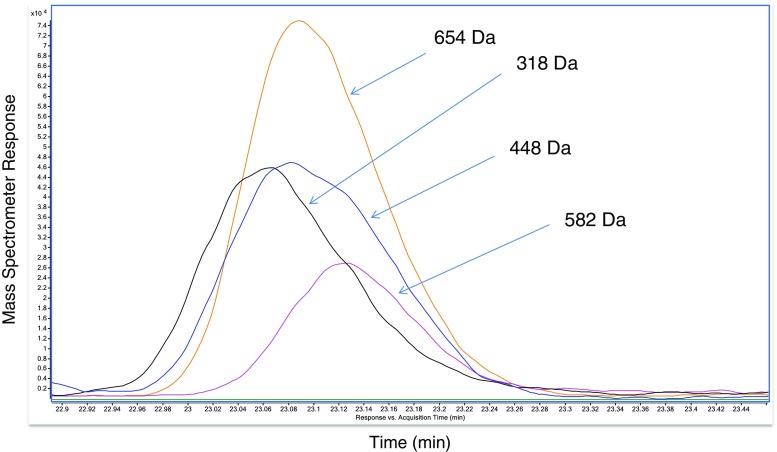
Comparison of mass chromatograms of analytes contained within CAD peak 37. Although the CAD cannot resolve these analytes, narrow mass chromatograms, labeled with nominal mass, from the high-resolution mass spectrometer clearly illustrate the presence of four distinct analytes, as opposed to multiple signals (fragments or adducts) from one compound

Some CAD peaks clearly contained more than one analyte. Usually, this was readily ascertained by comparing accurate (narrow) mass chromatograms, from signals observed in the summed mass spectrum, as shown in Fig. 5. This figure illustrates how the much faster data acquisition rate, effectively narrow detection window and specificity of TOF-HRMS, relative to the universal CAD, allowed confirmation of individual analytes. Other CAD peaks, indicated by “*” in the table, contained low-levels of other signals, observed in corresponding mass spectra, indicating the presence of multiple analytes. Based upon their relatively low MS signals, these were presumed to be present below the levels of interest, with respect to a threshold of toxicological concern safety evaluation.

No identification or useful characterization, from a safety assessment perspective, was obtained for about 10% of the total CAD signal. However, the data also indicate that no single unknown component exists above a level of 1.2% of the total CAD signal. For the purposes of a safety assessment such as threshold of toxicological concern, this would mean an unknown 1.2% constituent would be assigned the most conservative toxicity level for the overall material consideration.

Additional constituent information could have been obtained with this Gingko example, with further experimentation; however, from a safety standpoint, this was unnecessary. For instance, in some cases, MS/MS experiments were not performed to assist identifications (low level analytes or secondary experiments suggested after data interpretation). With additional effort, it is clear that further identifications could have been made, increased confidence on some identifications would be possible and perhaps improved specificity for some characterizations could be reported.

However, there would be a diminishing return on effort investment fairly quickly considering the level of data required for the safety assessment was largely met. This factor can be expected for each botanical characterization, by this or any other approach, and consideration when enough information has been gathered will be inherent to each project. This preliminary investigation was not designed to correlate quantitative CAD data to more rigorous quantitation using available authentic standards. Rather, the authentic standards were utilized for qualitative confirmation and were assayed at only one level. Future efforts will be designed for such a comparison, particularly at the CAD limit of detection.

It may be possible to make some assessment of the percentage of the total mass of the botanical that was detected by the CAD (assumed here to be 100%). It is possible that some measurable amount of material is lost due to volatility (under-represented or not detected by the CAD) or is not eluted (retained permanently or beyond the experiment duration) from the column. More remains to be learned about the broad chromatographic hump seen in some botanical analyses, such as this one, with respect to its relevance to the safety assessment.

In all probability, the most significant variables impacting overall effort, using this approach to characterize other botanicals, will be (1) the number of analytes present, (2) what is already known about that botanical and reported in the literature, and (3) what chromatography (and chromatographic method development) will be required.

### Botanical safety and quality assessments

Botanical extracts are complex mixtures of various chemical constituents and as such the chemical constituent identification approach has potential for application to the safety and quality assessment of botanicals. The U.S. Cosmetic Ingredient Review (CIR) considered safety data on chemical constituents in their favorable assessment of *Calendula officinalis* extracts [19]. Monitoring of marker chemical constituents is often used for quality assessment of botanical extracts. The analytical techniques presented in this paper have been used for safety assessments of other botanicals and these results, from a toxicology perspective, have been reported [17]. That paper discusses the relationships between dose and threshold of toxicological concern with quantitation of analytes and limits of quantitation detailed here. We have begun to utilize this approach for identification and quantitation of extractable constituents from other materials, as they relate to safety assessments. This work will be the subject of future reports.

## Electronic supplementary material

ESM 1 (PDF 881 kb)
