# Supplementary material for: A multi-detector chromatographic approach for characterization and quantitation of botanical constituents to enable in silico safety assessments
Source: Anal Bioanal Chem. 2018 Jul 11;410(21):5143–54. doi: 10.1007/s00216-018-1163-y (PMC6061755; doi:10.1007/s00216-018-1163-y)
Supplement: Supplementary file 1 — (PDF 881 kb) [file 216_2018_1163_MOESM1_ESM.pdf]

Analytical and Bioanalytical Chemistry

Electronic Supplementary Material

A multi-detector chromatographic approach for characterization and quantitation of botanical constituents to enable in silico safety assessments

Timothy R. Baker, Brian T. Regg

Table S1 Proposed identifications of the 83 CAD peaks observed during the analysis of the Spectrum Ginkgo extract

CAD Peak number	RT (min.)	+/- ion mode	Mass Accuracy (Score)	Proposed ID (CAS #)	Molecular Formula	CAD %	Structure	Comments	MS/MS Product Ions (m/z). Precursor not listed
1	0.94	Pos	-1.3 ppm (100)<br><br>-0.1 ppm (99)	Magnesium	Mg^{2+}	1.2 %		Magnesium clusters with acetonitrile and formic acid in positive ion mode and Magnesium clusters with formic acid in negative ion mode. Example here is $[MgFm+2ACN]^{+}$. MgFm2 repeat has a mass difference of 113.98043. In negative ion, example is $[Mg+3Fm]^{-}$. Isotope pattern consistent with Mg.	
2	1.00	Pos	-1.8 ppm<br>(100)	Choline<br>(67-48-1)	$C_5H_{14}N_1O_1$	3.1 %	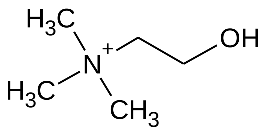	RT, UV and MS/MS spectra consistent with authentic standard. 193	60.0810 (+)<br>58.0654<br>45.0336
3	1.10	Pos	0.1 ppm<br>(88)	Trigonelline<br>(6138-41-6)	$C_7H_7N_1O_2$	4.1 %	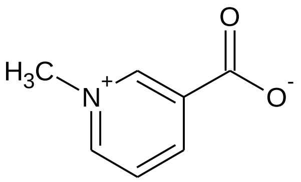	RT, UV and MS/MS spectra consistent with authentic standard.	110.0601 (+)<br>94.0632<br>92.0498<br>78.0342
		Pos	-1.8 ppm<br>(88)	L-Proline<br>(147-85-3)	$C_5H_9N_1O_2$			RT, UV and MS/MS spectra consistent with authentic standard.	70.0653 (+)<br>43.0542

							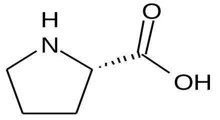		
		Pos	-1.8 ppm<br>(100)	L-Valine<br>(72-18-4)	$C_5H_{11}N_1O_2$		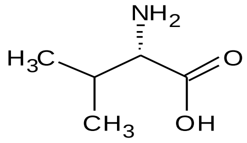	RT, UV and MS/MS spectra consistent with authentic standard.	72.0808 (+)<br>55.0540
		Neg	1.9 ppm<br>(98)	Mannitol<br>(69-65-8)	$C_6H_{14}O_6$		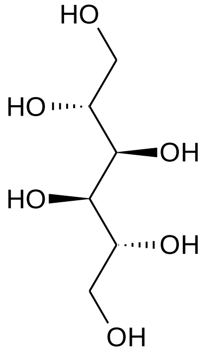	RT, UV and MS/MS spectra consistent with authentic standard.	163.0599 (-)<br>101.0239<br>89.0245<br>71.0138<br>59.0142
		Neg	0.8 ppm<br>(96)	Quinic Acid<br>(77-95-2)	$C_7H_{12}O_6$		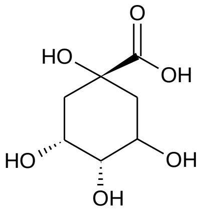	RT, UV and MS/MS spectra consistent with authentic standard.	127.0405 (-)<br>93.0351<br>85.0299<br>59.0142

4	1.23	Pos	0.6 ppm (98)	Ginkgotoxin-5-O-glucoside (323579-25-5)	$C_{15}H_{23}N_1O_8$	5.6%	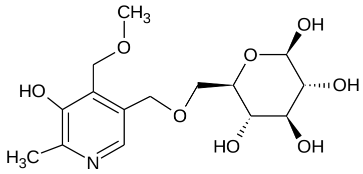	MS/MS indicates hexose and is consistent with structure.	314.1234 (+) 184.0969 152.0704 136.0755 108.0808
		Neg	0.3 ppm (100)	Unknown	$C_7H_{12}O_5$		?	Unknown, but probably similar to Quinic acid (less oxygen).	129.0557 (-) 111.0153 95.0504 73.0297
5	4.88	Pos/Neg	-0.5 ppm (95) 0.9 ppm (100)	Protocatechuic Acid (99-50-3)	$C_7H_6O_4$	0.7%	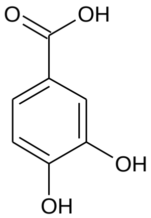	RT, UV and MS/MS spectra consistent with authentic standard.	109.0296 (-)
6	7.14	Pos/Neg	-0.6 ppm (100) 0.4 ppm (100)	Xanthurenic Acid (59-00-7)	$C_{10}H_7N_1O_4$	0.2 %	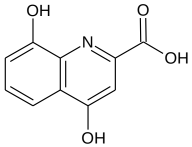	RT, UV and MS/MS spectra consistent with authentic standard.	188.0341 (+) 178.0497 160.0392 132.0442 104.0491

7	8.44	Pos/Neg	0.1 ppm<br>(99)<br>-0.4 ppm<br>(100)	Unknown	$C_{16}H_{24}O_9$	0.6%	?	Observed as $[M+NH_4]^+$ in positive ion mode and $[M+ Formate]^-$ in negative ion mode.	204.0304 (-)<br>160.0400
		Pos	-1.0 ppm<br>(98)	L-Tryptophan<br>(73-22-3)	$C_{11}H_{12}N_2O_2$		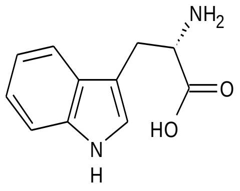	RT, UV and MS/MS spectra consistent with authentic standard.	188.0709 (+)<br>159.0918<br>146.0601<br>118.0653
8	10.39	Pos	-0.6 ppm<br>(98)	Caffeine<br>(58-08-2)	$C_8H_{10}N_4O_2$	0.3 %		RT, UV and MS/MS spectra consistent with authentic standard.	138.0664 (+)<br>110.0713<br>69.0447<br>42.0432
9	11.18	Pos/Neg	-0.3 ppm<br>(99)<br>-0.2 ppm<br>(100)	Gallocatechin<br>(3371-27-5)	$C_{15}H_{14}O_7$	3.4 %	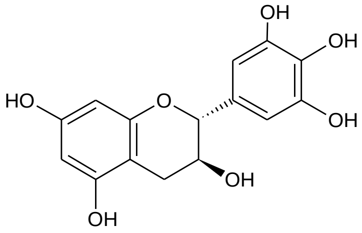	RT, UV and MS/MS spectra consistent with authentic standard.	261.0768 (-)<br>219.0661<br>179.0348<br>165.0189<br>125.0241

		Pos/Neg	0.6 ppm<br>(100)<br>0.3 ppm<br>(100)	Hydroxy-benzaldehyde	$C_7H_6O_2$		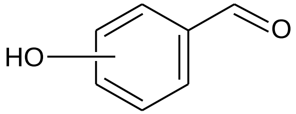	MS/MS spectrum suggests Hydroxybenzaldehyde	92.0267(-)<br>65.0397
10	11.76	Neg	0.1 ppm<br>(100)	Vanillic Acid<br>(121-34-6)	$C_8H_8O_4$	0.3 %	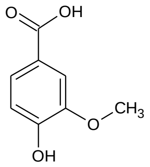	RT, UV and MS/MS spectra consistent with authentic standard.	152.0113 (-)<br>123.0451<br>108.0217<br>95.0140
		Neg	0.6 ppm<br>(87)	Esculetin<br>(305-01-1)	$C_9H_6O_4$		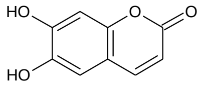	RT, UV and MS/MS spectra consistent with authentic standard.	149.0240 (-)<br>133.0295<br>105.0346<br>89.0398<br>77.0398
		Pos/Neg	-0.8 ppm<br>(100)<br>0.1 ppm<br>(100)	Catechin<br>(7295-85-4)	$C_{15}H_{14}O_6$		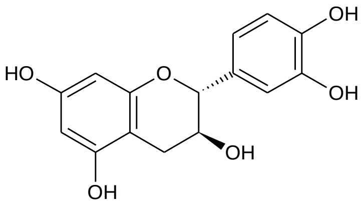	RT, UV and MS/MS spectra consistent with authentic standard.	249.0760 (+)<br>207.0653<br>165.0546<br>139.0390<br>123.0441

		Neg	0.5 ppm<br>(100)	Salicylic Acid<br>(69-72-7)	$C_7H_6O_3$		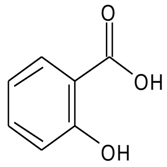	RT, UV and MS/MS spectra consistent with authentic standard.	93.0349 (-)<br>65.0397
		Pos/Neg	-0.5 ppm<br>(97)<br>-0.4 ppm<br>(100)	Quercetin with 2 glucose and 1 rhamnose	$C_{39}H_{50}O_{25}$		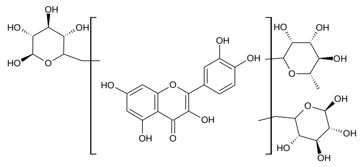	MS/MS shows losses of glucose and rhamnose.	757.1968 (+)<br>611.1613<br>465.1028<br>309.0971<br>147.0440
11	12.95	Pos/Neg	-0.6 ppm<br>(99)<br>-0.3 ppm<br>(99)	Unknown	$C_{19}H_{28}O_{11}$	0.3 %	?	Observed as $[M+NH_4]^+$ in positive ion mode and $[M+ Formate]^-$ in negative ion mode. MS/MS indicates hexose present.	431.1561 (-)<br>269.1030<br>161.0460<br>101.0245
12	13.43	Pos/Neg	-0.2 ppm<br>(99)<br>-0.7 ppm<br>(99)	Unknown	$C_{32}H_{44}O_{17}$	0.7 %	?	Observed as $[M+NH_4]^+$ in positive ion mode and $[M+ Formate]^-$ in negative ion mode. MS/MS indicates hexose present.	699.2510 (-)<br>537.1978<br>375.1446<br>195.0667<br>179.0719

13	14.17	Pos/Neg	-0.5 ppm (99) -0.6 ppm (99)	Kaempferol tetraglycoside (2 glucose and 2 rhamnose)	$C_{39}H_{50}O_{24}$	0.2 %	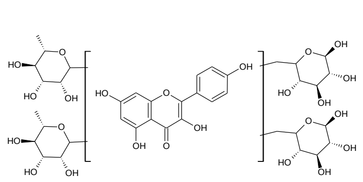	Proposed compound from MS/MS data. Connectivity unknown.	741.2040 (+) 595.16480 449.1074 287.0549 147.0439
14	14.64	Pos/Neg	-0.2 ppm (99) -0.8 ppm (100)	Glucopyranosyl rutin	$C_{33}H_{40}O_{21}$	0.2 %	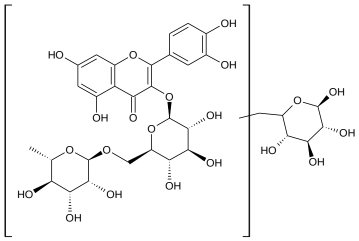	Proposed compound based on MS/MS data.	627.1558 (+) 611.1591 465.1032 303.0496 129.0552
		Pos/Neg	-0.5 ppm (100) -0.3 ppm (100)	Astilbin (29838-67-3)	$C_{21}H_{22}O_{11}$		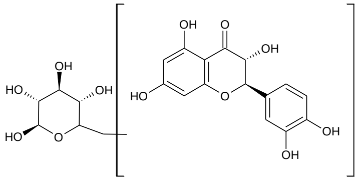	Proposed based on MS/MS data.	289.0711 (+) 271.0603 243.0661 153.0181 149.0225
15	15.34	Pos/Neg	-0.4 ppm (100) -1.2 ppm (93)	Kaempferol rhamnosyl glucoside	$C_{27}H_{30}O_{15}$	0.3 %	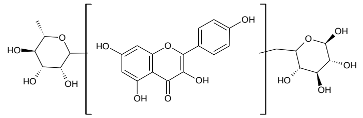	Proposed compound based on MS/MS data and literature [17]. Observed as [M+ Formate]$^-$ in negative ion mode.	449.1081 (+) 287.0557 129.0546

16	16.30	Pos/Neg	-0.1 ppm<br>(100)<br><br>-0.5 ppm<br>(99)	Bilobalide<br>(33570-04-6)	$C_{15}H_{18}O_8$	5.4 %	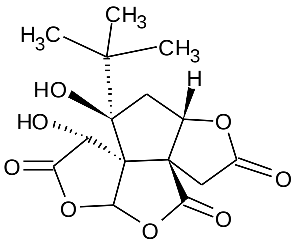	RT, UV and MS/MS spectra consistent with authentic standard.	309.0974 (+)<br>245.0822<br>235.0961<br>217.0862<br>191.1068<br>173.0964<br>111.0803<br>57.0702
17	16.60	Pos/Neg	-0.3 ppm<br>(100)<br><br>-0.9 ppm<br>(99)	Quercetin rhamnosyl rutinoside isomer	$C_{33}H_{40}O_{20}$	0.3 %	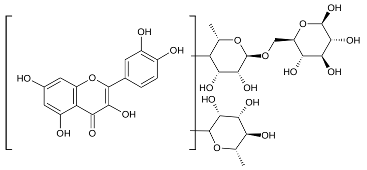	Proposed compound based on MS/MS and literature [17].	611.1607 (+)<br>595.1657<br>465.1027<br>449.1078<br>303.0503<br>287.0551
18	16.80	Pos/Neg	-0.7 ppm<br>(100)<br><br>-0.1 ppm<br>(99)	Kaempferol di-rhamnosyl-glucoside	$C_{33}H_{40}O_{19}$	0.9 %	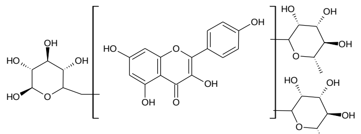	Proposed structure based on MS/MS and literature [18].	595.1657 (+)<br>449.1075<br>287.0552

		Pos/Neg	0.1 ppm<br>(100)<br>-0.2 ppm<br>(99)	Isorhamnetin di-glucosyl-rhamnoside	$C_{34}H_{42}O_{21}$		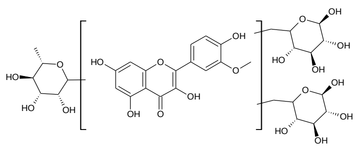	Proposed structure based on MS/MS.	641.1715 (+)<br>625.1776<br>479.1185<br>317.0659
19	17.18	Neg	-0.4 ppm<br>(100)	?	$C_{15}H_{20}O_{9}$	0.8 %	?	This unknown one of several analytes within this peak. *	299.1137 (-)<br>193.1234<br>181.1233<br>83.0502<br>72.9930
20	17.51	Pos/Neg	-0.9 ppm<br>(99)<br>-0.9 ppm<br>(99)	Quercetin rhamnosyl rutinoside isomer	$C_{33}H_{40}O_{20}$	0.7 %	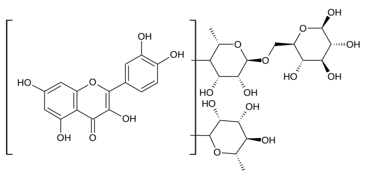	Proposed compound based on MS/MS.	611.1609 (+)<br>595.1652<br>465.1030<br>449.1079<br>303.0507<br>287.0554
		Pos/Neg	-0.8 ppm<br>(99)	Unknown	$C_{27}H_{30}O_{15}$		?	MS/MS shows some indication of a possible kaempferol rutinoside.	285.0405 (-)<br>284.0326<br>151.0058

			-0.8 ppm<br>(100)						
		Pos/Neg	-0.2 ppm<br>(99)<br>-0.2 ppm<br>(99)	Kaempferol glucosyl-coumaryl-glucosyl rhamnoside	$C_{48}H_{56}O_{27}$		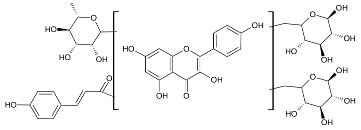	MS/MS fragments indicate loss of coumaryl, rhamnose and hexose.	901.2417 (-)<br>755.2047<br>609.1445<br>307.0822
21	17.86	Pos/Neg	-0.4 ppm<br>(100)<br>-0.7 ppm<br>(99)	Myricetin rutinoside	$C_{27}H_{30}O_{17}$	0.6 %	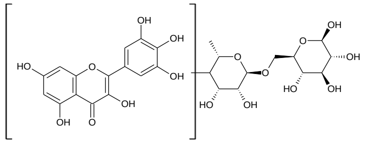	Proposed structure based on MS/MS and literature [17].	316.0224 (-)<br>178.9988
		Neg	-0.1 ppm<br>(100)	Bilobalide isomer	$C_{15}H_{18}O_{8}$		?	Probable isomer of Peak 16.	309.0977 (+)<br>245.0820<br>217.0863<br>191.1070<br>173.0961<br>111.0807

									57.0704
22	18.31	Pos/Neg	-0.7 ppm<br>(99)<br><br>-0.6 ppm<br>(100)	Quercetin rhamnosyl rutinoside isomer	$C_{33}H_{40}O_{20}$	0.9 %	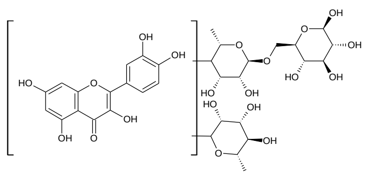	Proposed compound based on MS/MS and literature [17].	611.1607 (+)<br>595.1657<br>465.1027<br>449.1078<br>303.0503<br>287.0551
23	18.65	Pos/Neg	-0.2 ppm<br>(100)<br><br>-0.8 ppm<br>(99)	Quercetin rhamnosyl rutinoside isomer	$C_{33}H_{40}O_{20}$	0.5 %	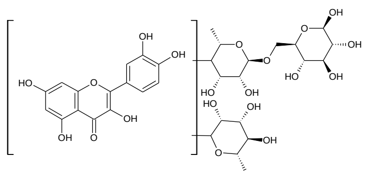	Proposed compound based on MS/MS and literature [17].	611.1609 (+)<br>595.1654<br>465.1025<br>449.1081<br>303.0502<br>287.0557
24	19.26	Pos/Neg	-0.6 ppm<br>(100)<br><br>-0.6 ppm<br>(100)	Rutin isomer	$C_{27}H_{30}O_{16}$	1.4 %	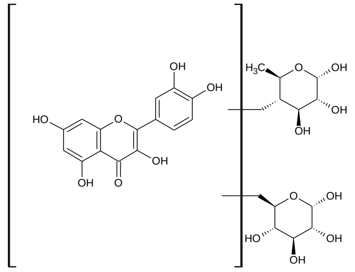	Proposed compound based on MS/MS and literature [17].	449.1080 (+)<br>287.05538<br>85.0282

25	19.51	Pos/Neg	-0.6 ppm<br>(98)<br>-0.5 ppm<br>(99)	Ginkgolide J<br>(107438-79-9)	$C_{20}H_{24}O_{10}$	0.3 %	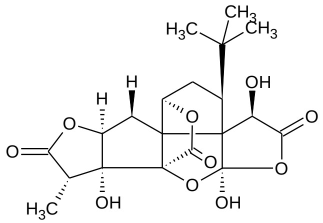	RT, UV and MS/MS spectra consistent with authentic standard. Observed as [M+ Formate]$^-$ in negative ion mode.	379.1387 (+)<br>351.1432<br>309.0606<br>235.0604<br>57.0699
26	19.72	Pos/Neg	-0.1 ppm<br>(98)<br>-0.5 ppm<br>(98)	Quercetin glucosyl-coumarylglucosyl rhamnoside	$C_{42}H_{46}O_{23}$	0.7 %	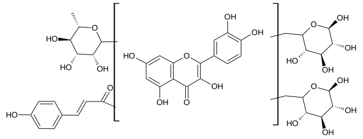	Proposed structure based on MS/MS and literature [17].	757.1964 (+)<br>611.1614<br>465.1028<br>309.0971<br>147.0439
27	19.78	Pos/Neg	-0.7 ppm<br>(99)<br>-0.6 ppm<br>(99)	Kaempferol di-rhamnosyl-glucoside	$C_{33}H_{40}O_{19}$	1.4 %	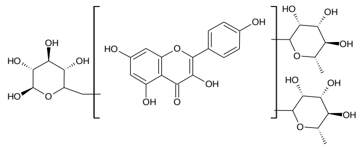	Proposed structure based on MS/MS. Kaempferol fragment ion observed in MS/MS.	595.1655 (+)<br>449.1074<br>287.0550<br>85.0285
28	20.12	Pos/Neg	-2.8 ppm<br>(95)<br>-2.1 ppm<br>(97)	Rutin<br>(153-18-4)	$C_{27}H_{30}O_{16}$	5.5 %	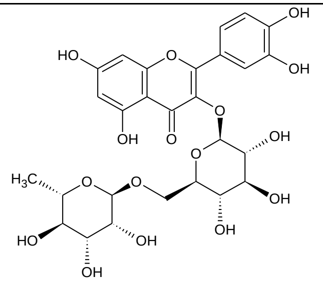	RT, UV and MS/MS spectra consistent with authentic standard. Possible second isomer nearly coeluting with Ruti1n.	465.1029 (+)<br>303.0507<br>129.0549<br>85.0288

		Pos/Neg	-0.3 ppm (97)<br>-0.5 ppm (99)	Ginkgolide C (15291-76-6)	$C_{20}H_{24}O_{11}$		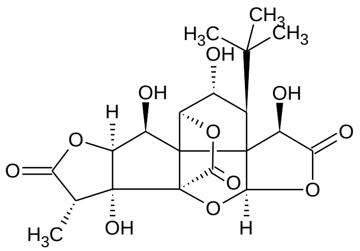	RT, UV and MS/MS spectra consistent with authentic standard. Relative amounts of these two coeluting compounds probably not different by more than a factor of two.	395.1345 (+)<br>377.1235<br>349.1281<br>325.0554<br>307.0451
29	20.50	Pos/Neg	-0.3 ppm (100)<br>-0.7 ppm (98)	Isoquercetin (482-35-9)	$C_{21}H_{20}O_{12}$	0.5 %	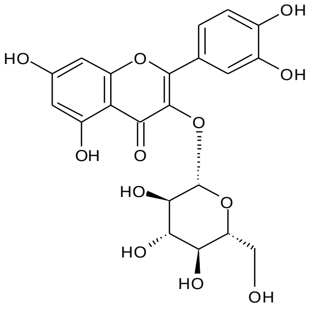	RT, UV and MS/MS spectra consistent with authentic standard.	301.0344 (-)<br>300.0275<br>271.0245<br>151.0034
		Pos/Neg	-0.2 ppm (100)<br>-0.3 ppm (100)	Laricitrin rutinoside Isomer	$C_{28}H_{32}O_{17}$		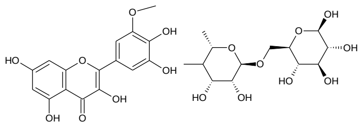	Proposed structure based on MS/MS and literature [17]. Isorhamnetin fragment observed in MS/MS spectrum.	495.1136 (+)<br>333.0610<br>85.0289
30	20.70	Pos/Neg	-0.2 ppm (100)<br>-0.7 ppm (99)	Laricitrin rutinoside Isomer	$C_{28}H_{32}O_{17}$	0.6 %	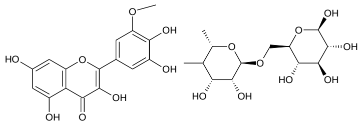	Proposed structure based on MS source fragments, literature [17].	495.1134 (+)<br>333.0608<br>85.0284

31	20.97	Pos/Neg	-0.4 ppm (99)<br>-0.3 ppm (99)	Kaempferol glucosylcoumarylglucosyl rhamnoside	$C_{42}H_{46}O_{22}$	0.2 %	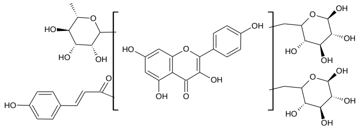	Proposed structure based on MS/MS and literature [17].	739.1881 (-)<br>593.1516<br>284.0324
32	21.14	Pos/Neg	-0.5 ppm (99)<br>-0.8 ppm (98)	Quercetin glucosylcoumarylglucosyl rhamnoside	$C_{42}H_{46}O_{23}$	1.0 %	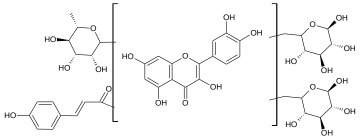	Proposed structure based on MS/MS and literature [17].	755.1827 (-)<br>609.1459<br>301.0338<br>300.0274<br>178.9980
		Pos/Neg	-0.1 ppm (100)<br>-0.4 ppm (99)	Unknown	$C_{26}H_{34}O_{11}$		?	Observed as $[M+NH_4]^+$ in positive ion mode and $[M+ Formate]^-$ in negative ion mode. MS/MS indicates hexose present.	359.1507 (-)<br>341.1395<br>329.1395<br>44.9983

33	22.09	Pos/Neg	-0.7 ppm<br>(10)<br><br>-0.8 ppm<br>(99)	Isomer of Rutin	$C_{27}H_{30}O_{16}$	1.3 %	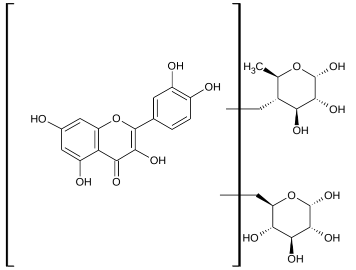	Structure similar to Rutin based on similar MS/MS fragment ions observed from Rutin. MS/MS spectrum shows aglycone quercetin fragment.	465.1031 (+)<br>303.0506
34	22.20	Pos/Neg	-0.4 ppm<br>(100)<br><br>-0.3 ppm<br>(98)	Kaempferol rutinoside<br>(17650-84-9)	$C_{27}H_{30}O_{15}$	3.8 %	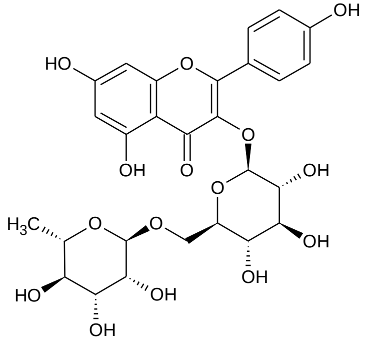	RT, UV and MS/MS spectra consistent with authentic standard.	449.1078 (+)<br>287.0554<br>129.0545<br>85.0285
35	22.63	Pos/Neg	-0.4 ppm<br>(99)<br><br>-0.1 ppm<br>(100)	Quercitrin<br>(522-12-3)	$C_{21}H_{20}O_{11}$	0.2 %	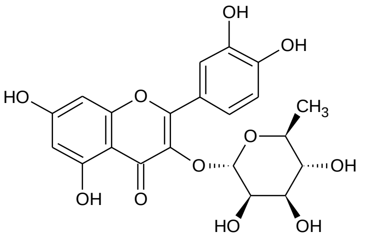	RT, UV and MS/MS spectra consistent with authentic standard.	303.0506 (+)

36	22.83	Pos/Neg	-0.3 ppm<br>(99)<br>-0.6 ppm<br>(99)	Isorhamnetin rutinoside<br>(604-80-8)	$C_{28}H_{32}O_{16}$	3.2 %	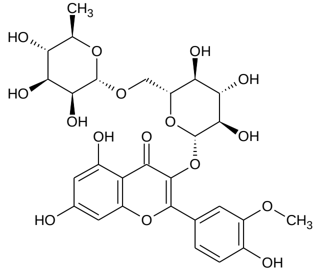	RT, UV and MS/MS spectra consistent with authentic standard.	315.0510 (-)<br>151.0029
37	23.16	Pos/Neg	-0.3 ppm<br>(99)<br>-0.6 ppm<br>(99)	Limocitrin rutinoside<br>(489-33-8)	$C_{29}H_{34}O_{17}$	0.5 %	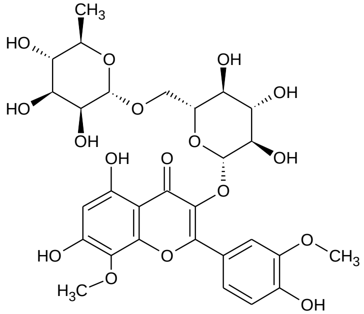	Possibly Limocitrin (489-33-8) rutinoside. MS/MS indicates rutinoside and limocitrin aglycone.	347.0762 (+)<br>85.0284
		Pos/Neg	-0.1 ppm<br>(100)<br>-0.5 ppm<br>(100)	Kaempferol glucoside isomer	$C_{21}H_{20}O_{11}$		?	MS/MS indicates presence of hexose.	287.0551 (+)<br>153.0184
		Pos/Neg	-0.1 ppm<br>(100)<br>-0.5 ppm<br>(100)	Myricetin<br>(529-44-2)	$C_{15}H_{10}O_8$		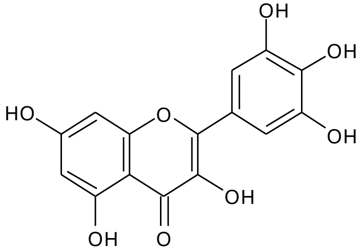	RT, UV and MS/MS spectra consistent with authentic standard.	178.9982 (-)<br>151.0033<br>137.0243<br>109.0294

									107.0190
38	23.38	Pos/Neg	0.1 ppm<br>(99)<br>-1.0 ppm<br>(97)	Genistein rutinoside	$C_{27}H_{30}O_{14}$	1.8 %	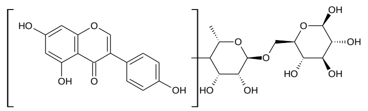	MS/MS suggests genistein aglycone, hexose and rhamnose (cannot rule out apigenin as the aglycone). This appears to be the more abundant of the two similar coeluting compounds.	431.0986 (-)<br>268.0377
		Pos/Neg	-0.4 ppm<br>(100)<br>-0.4 ppm<br>(100)	Isorhamnetin glucoside<br>(5041-82-7)	$C_{22}H_{22}O_{12}$		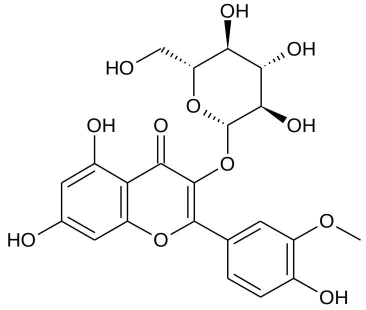	RT, UV and MS/MS spectra consistent with authentic standard.	317.0659 (+)<br>209.1537
39	23.82	Pos/Neg	1.3 ppm<br>(98)<br>-3.7 ppm<br>(92)	Unknown	$C_{28}H_{36}O_{13}$	0.9 %	?	Observed as $[M+NH_4]^+$ in positive ion mode. MS/MS indicates presence of hexose.	535.2755(-)<br>417.1554<br>181.0503
40	24.20	Pos/Neg	-0.2 ppm	Isomer of Kaempferol	$C_{27}H_{30}O_{15}$	2.3 %		MS/MS suggests hexose and kaempferol aglycone.	433.1132 (+)<br>287.0553

			(100)<br><br>0.4 ppm<br>(99)	rutinoside<br>(17650-84-9)			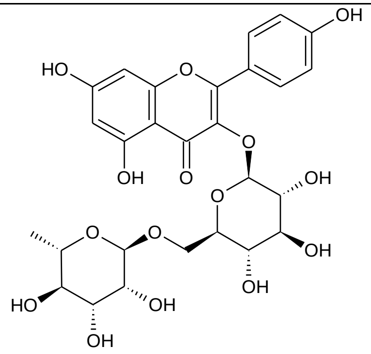		147.0649<br>129.0546<br>85.0285
		Pos/Neg	-0.2 ppm<br>(100)<br><br>-0.5 ppm<br>(100)	Kaempferol rutinoside substructure	$C_{43}H_{46}O_{22}$		?	MS/MS data confirms substructure of kaempferol rutinoside. Remainder of formula corresponds to epigallocatechin methyl ether.	629.2055 (+)<br>611.1949<br>483.1508<br>287.0555<br>147.0442
41	24.75	Pos/Neg	-0.5 ppm<br>(100)<br><br>-0.5 ppm<br>(100)	Isomer of<br>Quercitrin<br>(522-12-3)	$C_{21}H_{20}O_{11}$	0.8 %	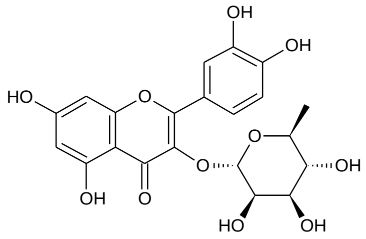	MS/MS confirms quercetin aglycone and rhamnose.	303.0509 (+)
		Pos/Neg	-0.4 ppm<br>(99)	Unknown	$C_{26}H_{34}O_{10}$		?	Observed as $[M+NH_4]^+$ in positive ion mode and $[M+ Formate]^-$ in negative ion mode.	521.2032 (-)<br>503.1925

			-0.5 ppm<br>(100)						491.1922
42	24.82	Pos/Neg	-0.4 ppm<br>(99)<br>-0.5 ppm<br>(100)	Unknown	$C_{32}H_{42}O_{14}$	0.3 %	?	This empirical formula is confident, but MS/MS does not clarify structure.	621.2560 (+)<br>456.2129<br>426.2025<br>382.2122<br>259.1442
43	25.21	Pos/Neg	-1.2 ppm<br>(81)<br>-5.4 ppm<br>(70)	Kaempferol rhamnoside	$C_{21}H_{20}O_{10}$	0.3 %	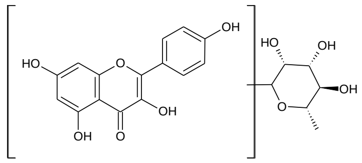	MS/MS confirms kaempferol aglycone and rhamnose.	285.0399 (-)<br>284.0327<br>255.0301<br>227.0346
		Pos/Neg	-0.1 ppm<br>(99)<br>-0.4 ppm<br>(100)	Unknown	$C_{20}H_{36}O_{11}$		?	Observed as $[M+NH_4]^+$ in positive ion mode and $[M+ Formate]^-$ in negative ion mode. MS/MS confirms hexose.	451.2187 (-)<br>289.1649<br>161.0452<br>101.0241<br>71.0144

44	25.44	Pos/Neg	-1.0 ppm (98) 0.6 ppm (99)	Ginkgolide Isomer	$C_{20}H_{24}O_{10}$	0.4 %	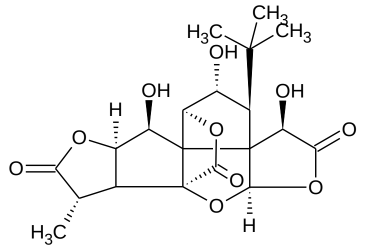	Possibly Ginkgolide M. Also observed as $[M+NH_4]^+$ in positive ion mode and $[M+ Formate]^-$ in negative ion mode. MS/MS spectrum similar to that of isobaric Ginkgolide B (Peak 46)	361.1302(+) 343.1157 305.1021 275.0926 113.0976
45	25.85	Pos/Neg	-0.5 ppm (99) -0.2 ppm (99)	Quercetin coumarylglucosyl rhamnoside isomer	$C_{36}H_{36}O_{18}$	4.1 %	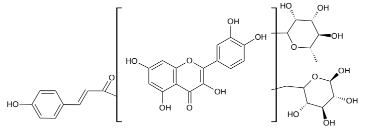	Assignment based on literature [17] and MS/MS in negative ion mode. MS/MS suggests coumaryl group, rutinoside and quercetin.	609.1465(-) 300.0274 178.9982
46	26.02	Pos/Neg	-1.1 ppm (98) -0.1 ppm (100)	Ginkgolide A (15291-75-5)	$C_{20}H_{24}O_9$	9.3 %	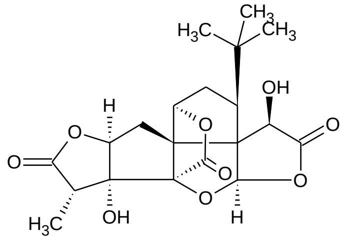	RT, UV and MS/MS spectra consistent with authentic standard. Also observed as $[M+NH_4]^+$ in positive ion mode and $[M+ Formate]^-$ in negative ion mode.	363.1436 (+) 345.1334 327.1226 299.1279 249.0757

		Pos/Neg	0.2 ppm<br>(100)<br>-1.5 ppm<br>(99)	Ginkgolide B<br>(15291-77-7)	$C_{20}H_{24}O_{10}$		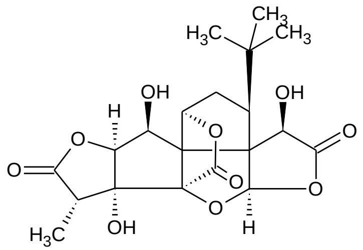	RT, UV and MS/MS spectra consistent with authentic standard. Also observed as $[M+NH_4]^+$ in positive ion mode and [M+ Formate]$^-$ in negative ion mode.	361.1280 (+)<br>343.1179<br>305.1018<br>257.0810<br>113.0959
47	26.41	Pos/Neg	-1.0 ppm<br>(99)<br>-0.7 ppm<br>(96)	Unknown	$C_{21}H_{30}O_9$	0.8 %	?	Observed as $[M+NH_4]^+$ in positive ion mode and [M+ Formate]$^-$ in negative ion mode. MS/MS indicates hexose.	245.1184 (-)<br>179.0559<br>119.0648<br>89.0245<br>59.0139
48	26.57	Pos	-0.8 ppm<br>(100)	Unknown	$C_{19}H_{22}O_5$	0.9 %	?	MS/MS shows facile loss of formic acid.	313.1418 (+)<br>285.1120<br>255.1064<br>151.0751<br>137.0595
49	27.71	Pos/Neg	-1.1 ppm<br>(99)<br>0.1 ppm	Quercetin<br>(117-39-5)	$C_{15}H_{10}O_7$	7.0 %	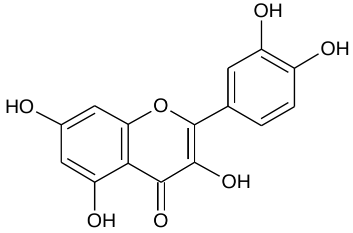	RT, UV and MS/MS spectra consistent with authentic standard.	273.0403 (-)<br>178.9983<br>151.0033<br>121.0294

			(100)						107.0139
50	27.78	Pos/Neg	-0.4 ppm<br>(100)<br>-0.6 ppm<br>(99)	Kaempferol coumarylglucosyl rhamnoside isomer	$C_{36}H_{36}O_{17}$	2.8 %	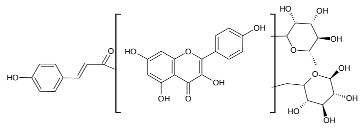	MS/MS confirms coumaryl, rhamnosyl hexosyl and kaempferol aglycone. Assignment also based on literature [17].	579.1517 (+)<br>433.1132<br>309.0969<br>287.0551<br>147.0440
51	28.61	Pos/Neg	-0.9 ppm<br>(100)<br>-0.6 ppm<br>(70)	Quercetin coumarylglucosyl rhamnoside isomer	$C_{36}H_{36}O_{18}$	0.9 %	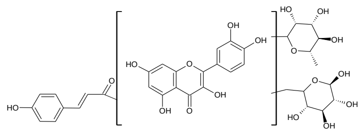	MS/MS confirms coumaryl, rhamnosyl hexosyl and quercetin aglycone. Assignment also based on literature [17].	609.1462(-)<br>300.0278<br>178.9981
52	29.20	Pos/Neg	0.3 ppm<br>(99)<br>0.7 ppm<br>(99)	Kaempferol-quercetin coumarylglucosyl rhamnoside isomer	$C_{72}H_{72}O_{35}$	0.8 %	Unknown combination of Peaks 50 and 51.	Appears to be chromatographically distinct dimer of MW = 740 and MW = 756 compound (Peaks 50 and 51). Observe doubly charged ion in both negative and positive ion modes. MS/MS confirms kaempferol and quercetin aglycones,rhamnose, glucose	1209.3260 (-)<br>901.2158<br>755.1821<br>609.1455<br>300.0271
53	29.55	Neg	-0.6 ppm<br>(100)	Unknown	$C_{21}H_{34}O_9$	0.2 %	?	Proposed elemental formula based on negative ion data. MS/MS indicates hexose.	249.1489 (-)<br>205.1603

									179.0557<br>101.0245<br>89.0240
54	29.78	Pos/Neg	0.2 ppm<br>(99)<br>-0.7 ppm<br>(100)	Kaempferol-quercetin coumarylglucosyl rhamnoside isomer	$C_{72}H_{72}O_{35}$	0.9 %	Unknown combination of Peaks 50 and 51.	Appears to be chromatographically distinct dimer of MW = 740 and MW = 756 compound (Peaks 50 and 51). Observe doubly charged ion in both negative and positive ion modes. MS/MS confirms kaempferol and quercetin aglycones,rhamnose, glucose	1209.3280 (-)<br>901.2191<br>755.1849<br>609.1455<br>300.0265
55	29.90	Pos/Neg	0.3 ppm<br>(100)<br>-0.5 ppm<br>(100)	Kaempferol-quercetin coumarylglucosyl rhamnoside isomer	$C_{72}H_{72}O_{35}$	0.9 %	Unknown combination of Peaks 50 and 51.	Appears to be chromatographically distinct dimer of MW = 740 and MW = 756 compound (Peaks 50 and 51). Observe doubly charged ion in both negative and positive ion modes. MS/MS confirms kaempferol and quercetin aglycones,rhamnose, glucose	1209.3316 (-)<br>901.2190<br>755.1831<br>609.1471<br>300.0288
56	30.36	Pos/Neg	-0.3 ppm<br>(100)<br>-0.6 ppm<br>(94)	Kaempferol coumarylglucosyl rhamnoside isomer dimer	$C_{72}H_{72}O_{34}$	0.8 %	Unknown dimer of Peak 50.	Appears to be chromatographically distinct dimer of MW = 740 (Peak 50). Observe doubly charged ion in both negative and positive ion modes. MS/MS confirms kaempferol aglycone, rhamnose, hexose.	1267.2979 (-)<br>1193.3358<br>981.2527<br>593.1520<br>285.0407

57	30.49	Pos/Neg	-0.8 ppm<br>(100)<br>-0.4 ppm<br>(100)	Kaempferol coumarylglucosyl rhamnoside isomer	$C_{36}H_{36}O_{17}$	0.6 %	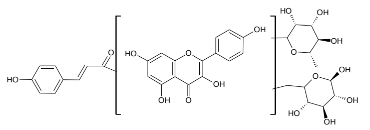	MS/MS confirms coumaryl, rhamnosyl hexosyl and kaempferol aglycone. Assignment also based on literature [17].	593.1519 (-)<br>413.0881<br>284.0330<br>145.0297
58	30.79	Pos/Neg	-0.6 ppm<br>(100)<br>-0.5 ppm<br>(100)	Genistein<br>(446-72-0)	$C_{15}H_{10}O_5$	1.5 %	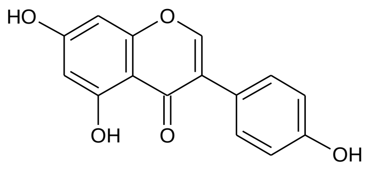	RT, UV and MS/MS spectra consistent with authentic standard.	243.0654 (+)<br>215.0705<br>197.0599<br>153.0182<br>91.0545
59	31.23	Pos/Neg	-0.1 ppm<br>(100)<br>-0.5 ppm<br>(100)	Kaempferol coumarylglucosyl rhamnoside isomer dimer	$C_{72}H_{72}O_{34}$	0.2 %	Unknown dimer of Peak 50.	Appears to be chromatographically distinct dimer of MW = 740 (Peak 50). Observe doubly charged ion in both negative and positive ion modes. MS/MS confirms kaempferol aglycone, rhamnose, hexose.	1193.3378 (-)<br>981.2531<br>739.1896<br>593.1533<br>285.0406
60	31.81	Pos/Neg	-0.9 ppm<br>(100)<br>-0.4 ppm<br>(100)	Kaempferol<br>(520-18-3)	$C_{15}H_{10}O_6$	3.6 %	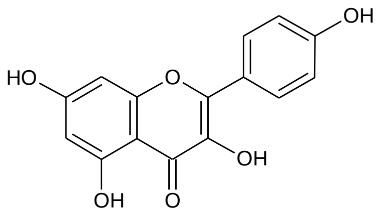	RT, UV and MS/MS spectra consistent with authentic standard.	258.0524 (+)<br>213.0542<br>153.0180<br>121.0285<br>68.9969

61	32.50	Pos/Neg	0.3 ppm<br>(99)<br>-0.3 ppm<br>(97)	Kaempferol coumarylglucosyl rhamnoside isomer dimer	$C_{72}H_{72}O_{34}$	0.3 %	Unknown dimer of Peak 50.	Chromatographically distinct dimer of MW = 740 (Peak 50). Observe doubly charged ion in both negative and positive ion modes. MS/MS confirms kaempferol aglycone, rhamnose, hexose.	1193.3355 (-)<br>739.1892<br>593.1507<br>285.0395
62	32.82	Pos/Neg	-0.2 ppm<br>(100)<br>-0.3 ppm<br>(100)	Isorhamnetin<br>(480-19-3)	$C_{16}H_{12}O_7$	0.4 %	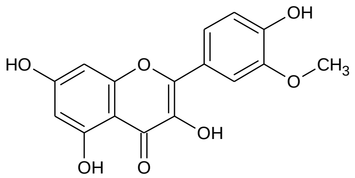	RT, UV and MS/MS spectra consistent with authentic standard.	302.0421 (+)<br>274.0469<br>229.0490<br>153.0180<br>93.0332
		Pos/Neg	0.2 ppm<br>(100)<br>-0.3 ppm<br>(100)	Kaempferol coumarylglucosyl rhamnoside isomer dimer	$C_{72}H_{72}O_{34}$		Unknown dimer of Peak 50.	Appears to be chromatographically distinct dimer of MW = 740 (Peak 50). Observe doubly charged ion in both negative and positive ion modes. MS/MS confirms kaempferol aglycone, rhamnose, hexose.	1267.3015 (-)<br>1193.3355<br>981.2521<br>593.1516<br>285.0396
		Pos/Neg	-0.3 ppm<br>(94)<br>-1.0 ppm	Unknown	$C_{21}H_{32}O_8$		?	Observed as $[M+NH_4]^+$ in positive ion mode and $[M+ Formate]^-$ in negative ion mode.	231.1374 (-)<br>161.0449<br>113.0246<br>101.0244

			(98)						89.0244
63	38.28	Pos/Neg	-0.5 ppm<br>(100)<br><br>-0.1 ppm<br>(100)	Unknown	$C_{16}H_{32}O_4$	0.3 %	?	Water loss fragment in positive ion mode suggest hydroxylated C16 chain acid.	269.2116 (-)<br>241.2714<br>141.1285<br>99.0816
64	40.60	Pos/Neg	-0.6 ppm<br>(97)<br><br>-0.6 ppm<br>(99)	Unknown	$C_{48}H_{76}O_{18}$	0.5 %	?	Observe $[M+NH4]^+$ in positive ion mode. DBE = 11. Appears to be triterpenoid saponin related structure. Possibly Dehydrosoyasaponin I.	731.4362 (-)<br>613.3739<br>523.3786<br>205.0713<br>163.0609
65	42.85	Pos	-0.7 ppm<br>(90)	Unknown	$C_{15}H_{22}O_3$	0.1 %	?	MS/MS response too weak. No MS/MS fragments.	
66	43.40	Pos	-1.0 ppm<br>(100)	(Z)-Alpha-Atlantone<br>(56192-70-2)	$C_{15}H_{22}O_1$	0.1 %	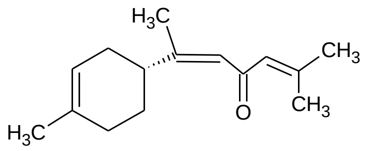	Proposed compound based on literature references and consistent with MS/MS spectrum.	177.1267 (+)<br>151.1116<br>109.0646<br>85.0649<br>57.0699
		Pos/Neg	0.7 ppm	Unknown	$C_{47}H_{74}O_{17}$		?	Observed as $[M+NH_4]^+$ in positive ion mode. Appears to be	569.3833 (-)

			(91)<br><br>-0.3 ppm<br>(99)					triterpenoid saponin related structure.	569.3833<br>523.3793<br>455.3543<br>143.0348<br>131.0244
67	44.27	Pos/Neg	-0.6 ppm<br>(82)<br><br>-0.4 ppm<br>(96)	Unknown	$C_{21}H_{40}O_7$		?	Also observed $[M+Na]^+$ and $[M+NH_4]^+$ in positive ion mode and observed $[M+ Formate]^-$ in negative ion mode.	387.2721 (+)<br>327.2528<br>309.2405<br>235.2062<br>217.1953
68	44.92	Pos/Neg	-0.5 ppm<br>(100)<br><br>-0.8 ppm<br>(99)	Soyasaponin I<br>(51330-27-9)	$C_{48}H_{78}O_{18}$	0.2 %	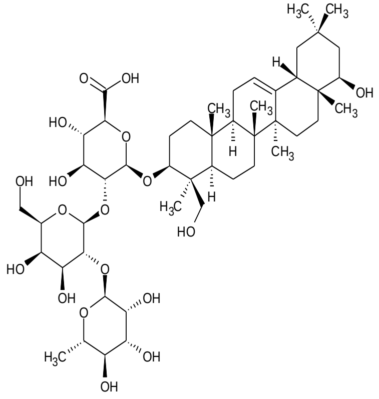	RT, UV and MS/MS spectra consistent with authentic standard.	733.4554 (-)<br>615.3903<br>525.3946<br>205.0715<br>163.0612
69	45.86	Pos/Neg	-0.8 ppm<br>(99)	Unknown	$C_{42}H_{68}O_{14}$	0.1 %	?	Similar structure to Peak 68 based on MS/MS. Appears to be triterpenoid saponin related	615.3904 (-)<br>457.3709

			-0.7 ppm (99)					structure.	157.0138 101.0244 89.0244
70	46.23	Pos/Neg	-0.8 ppm (99) -0.6 ppm (99)	Unknown	$C_{42}H_{68}O_{14}$	0.3 %	?	Similar structure to Peak 69 based on MS/MS. Appears to be triterpenoid saponin related structure.	615.3957 (-) 457.3705 157.0146 101.0245 89.0246
71	46.78	Pos/Neg	-0.4 ppm (99) -0.6 ppm (100)	Unknown	$C_{48}H_{78}O_{17}$	0.4 %	?	Observe $[M+NH4]^+$ in positive ion mode. DBE = 10. Appears to be triterpenoid saponin related structure.	717.4597 (-) 599.3963 509.3993 205.0711 163.0606
72	47.22	Pos/Neg	-0.8 ppm (100) -0.7 ppm (100)	Trihydroxy-octadecenoic acid	$C_{18}H_{34}O_5$	0.2 %	Connectivity unknown	MS/MS clearly suggests presence of acetate and three aliphatic hydroxyl groups. Position of hydroxyl groups and double bond unknown.	287.2225 (-) 269.2120 59.0140
73	48.37	Pos/Neg	-0.6 ppm	Unknown	$C_{42}H_{68}O_{13}$	0.2 %	?	Observe $[M+NH4]^+$ in positive ion mode. Appears to be triterpenoid	599.3955 (-)

			(100)<br><br>-0.8 ppm<br>(100)					saponin related structure.	509.4005<br>439.3582<br>113.0246<br>101.0245
74	52.68	Pos/Neg	-0.6 ppm<br>(100)<br><br>-0.4 ppm<br>(100)	Unknown	$C_{25}H_{40}O_5$	0.2 %	?	MS/MS suggests coumaryl group and dihydroxy aliphatic C16 acid.	163.0399 (-)<br>145.0293<br>119.0500<br>93.0349
75	54.62	Pos/Neg	-0.8 ppm<br>(98)<br><br>-0.8 ppm<br>(100)	Unknown	$C_{33}H_{56}O_{14}$	0.3 %	?	Observe $[M+NH4]^+$ in positive ion mode and $[M+ Formate]^-$ in negative ion mode.	415.1457 (-)<br>397.1354<br>277.2177<br>235.0821<br>89.0245
76	71.03	Pos	-2.9 ppm<br>(97)	Unknown	$C_{18}H_{30}O_2$	0.1 %	?	MS/MS suggests dihydroxy-aliphatic C18 chain.	261.2213 (+)<br>243.2106
77	73.79	Pos	-2.1 ppm<br>(97)	Unknown	$C_{30}H_{48}O_1$	0.2 %	?	Possible triterpene compound with single hydroxyl group. Very weak. MS/MS signal.	407.3676 (+)
78	75.84	Pos	-1.0 ppm	Unknown	$C_{18}H_{32}O_2$	0.1 %	?	Low Score caused by mass interferences and low signal.	

			(48)					Signal too weak for MS/MS. Possibly Linoleic acid.	
79	80.04	Not Detected	N/A	Unknown	Unknown	0.3 %	?	No discernable MS signal obtained for CAD peak.	
80	83.27	Pos/Neg	-0.7 ppm (92) -0.2 ppm (99)	Ginkgolic Acid (C13:0) (20261-38-5)	$C_{20}H_{32}O_3$	0.3 %	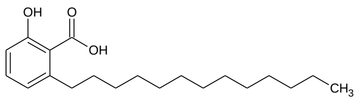	RT, UV and MS/MS spectra consistent with authentic standard.	275.2382 (-) 119.0502 106.0425
81	84.40	Pos/Neg	-0.9 ppm (97) -0.3 ppm (100)	Ginkgolic Acid (C15:1) (22190-60-7)	$C_{22}H_{34}O_3$	0.6 %	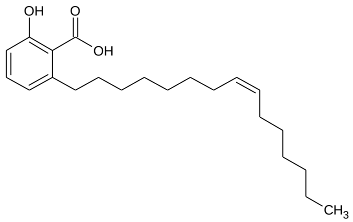	RT, UV and MS/MS spectra consistent with authentic standard.	301.2539 (-) 173.0662 119.0505 106.0427
82	87.57	Not Detected	N/A	Unknown	Unknown	0.3 %	?	No discernable MS signal obtained for CAD peak.	
83	105.72	Not Detected	N/A	Unknown	Unknown	0.1 %	?	No discernable MS signal obtained for CAD peak.	

* Mass Spectra from these CAD peaks also contained other lower level signals that indicate the presence of other analytes not reported.

Table S2 Additional Instrumental Conditions. All values as indication by the instrument

ESI (Agilent 6540)

- Capillary voltage – 4000 V
- Nozzle Voltage – 2000 V
- N_2 Drying Gas temperature – 350 °C
- Vaporizer Sheath Gas Temperature – 295 °C
- Nebulizer - 20 psig N_2
- Sheath Gas -12 L N_2/min
- Drying Gas – 5 L N_2/min
- Mass Range – m/z 50-1700, 2 scans/second

APCI

- Capillary voltage – 3500 V
- N_2 Drying Gas temperature – 350 °C
- APCI Vaporizer Temperature – 450 °C
- Nebulizer - 60 psig N_2
- Drying Gas – 5 L N_2/min

CAD

- Corona Current 4000 nA
- Gas – N2 35 psi. Range – 100 pA. Data collection rate -10 Hz
-

Fragmentation collision energy was optimized for each compound within the range of 8-50 eV
